# Green bioconversion of insoluble chitin: chitinase development pathways via multi-strategy synergy

**DOI:** 10.1186/s40643-025-00984-4

**Published:** 2026-01-27

**Authors:** Zhi-Ping Sai, Yi-Rui Yin, Li-Quan Yang, Jia-Hui Wang, Xin-Yi Yang, Fu-Xian Liu, Xin Jing, Yi Zhang, Yu-Da Li, Peng Sang, Zheng-Feng Yang

**Affiliations:** 1 College of Agriculture and Biological Science, Dali University, Dali, 671003 China; 2 Key Laboratory of Bioinformatics and Computational Biology, Department of Education of Yunnan Province, Dali University, Dali, 671003 China

**Keywords:** Insoluble chitin, Chitinase, Enzyme engineering, Bioconversion, AI-assisted design

## Abstract

As one of the most abundant natural polysaccharides on Earth, chitin is limited in its high-value utilization by its natural insolubility and high crystalline structure. Enzymatic degradation—especially via chitinases—serves as a highly promising approach for the green bioconversion of insoluble chitin. This review systematically analyzes the structural barriers that hinder the degradation of insoluble chitin and elucidates the enzymatic hydrolysis mechanisms underlying its conversion. Recent advances in enhancing chitinase catalytic efficiency through protein engineering approaches—including directed evolution, rational design, and domain fusion—are comprehensively discussed. In addition, the review highlights the multi-strategy synergistic frameworks that integrate AI-assisted enzyme design, immobilization technology, and expression regulation to achieve high-performance chitin bioconversion, which is intended to provide valuable references for the efficient bioconversion and resource recycling of insoluble chitin.

## Introduction

Chitin, the second most abundant natural polysaccharide on Earth after cellulose(Kumari et al. 2023), is a linear polymer composed of β-1,4-linked N-acetyl-D-glucosamine (GlcNAc) units. It is widely distributed in fungal cell walls, insect exoskeletons, and the exoskeletons of crustacean marine organisms(Majengbasan et al. 2025). With the global population growth and the rapid expansion of aquaculture, approximately 8 million tons of crustacean waste (e.g. crab, shrimp, and lobster shells) and over 10 million tons of mollusk shell waste are generated annually worldwide(Topić et al. 2023). These waste materials contain 20–30% chitin by dry weight, along with other high-value components such as proteins and astaxanthin(Ngasotter et al. 2022). However, improper disposal of these wastes poses significant risks of soil and water pollution, malodor emission, and landfill overloading, leading to severe environmental challenges(Ngasotter et al. 2023).

Chitin and its derivatives have attracted growing attention as sustainable biomaterials. Early studies showed that their hydrolysis products—mainly chitooligosaccharides (COSs) and chitosan oligosaccharides—exhibit antioxidant, antibacterial, antitumor, and immunostimulatory activities (Kumari et al. 2022; Anil et al. 2022). Later research confirmed their excellent biocompatibility, permeability, and biodegradability, expanding their applications across multiple fields (Piekarska et al. 2023).In biomedicine, chitin-based materials are used for drug delivery and tissue engineering (Dong et al. 2024; Rana et al. 2024). Their anti-inflammatory and antibacterial properties support cosmetic applications (Kodolova et al. 2023; Kulka et al. 2023), while biodegradability enables use in wastewater treatment (Bhatt et al. 2023) and bioethanol production (Huang et al. 2024). In food packaging, chitin composites enhance preservation and material performance (Wang et al. 2025a, b). Recent studies have further expanded chitin hydrolysate utilization into nanotechnology and materials science (Tsivileva et al. 2021; Fotodimas et al. 2025). Enzymatic control of degradation allows fabrication of tunable, biocompatible nanomaterials. Overall, transforming chitinous waste into high-value products through green biotechnologies represents an effective approach for sustainable resource utilization and environmental protection.

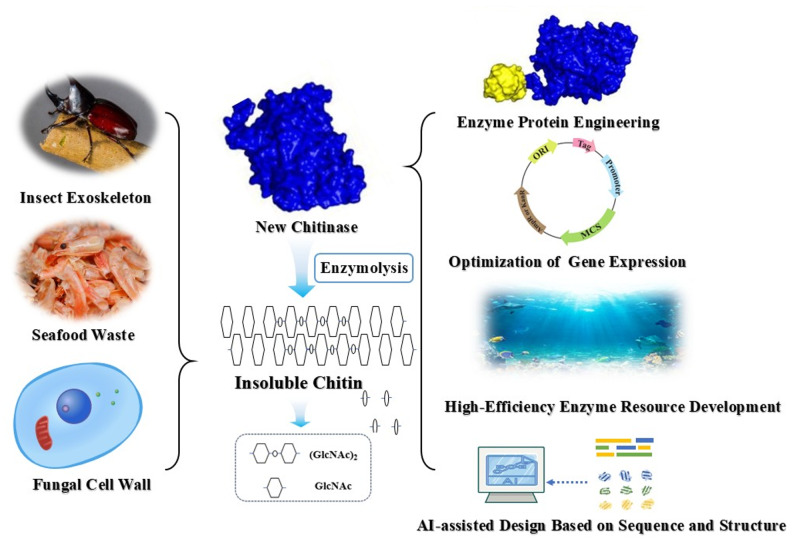



However, the primary form of natural chitin—insoluble crystalline chitin—remains a core bottleneck for its utilization. Its dense crystalline structure, highly ordered three-dimensional network, and insolubility in conventional solvents severely limit the development and commercial application of chitin(He et al. 2022). Although numerous studies have focused on chitin degradation, most rely on pretreated colloidal chitin as the substrate(He et al. 2022; Liu et al. 2024a, b; Majengbasan et al. 2025)—a limitation, as colloidal chitin production itself is constrained by high resource and cost requirements, involving multiple complex steps(Wang et al. 2023a, b, c). For instance, chitin raw materials (e.g. crab or shrimp shells) require extensive energy input for mechanical grinding; concentrated acids are used to dissolve chitin, followed by extensive washing to neutralize residual acids during precipitation(Xu et al. 2024; Matroodi et al. 2024)—a process that consumes large volumes of water, generates acidic wastewater, increases production costs, and poses additional environmental risks. Other pretreatment methods, such as TEMPO oxidation-ultrasonication and high-pressure homogenization(Ngasotter et al. 2022; Sampath et al. 2022), also suffer from high energy consumption or severe pollution. As eco-friendly catalysts that specifically hydrolyze β-1,4-glycosidic bonds, chitinases have emerged as a research focus in recent years for insoluble chitin conversion. Compared with traditional physical methods(Zhai et al. 2020) (high energy consumption) and chemical methods(Günal et al. 2025) (severe pollution), enzymatic degradation of insoluble chitin offers distinct advantages: mild reaction conditions, high substrate specificity, good environmental compatibility(Sagar et al. 2025), and no toxic wastewater discharge(Cao et al. 2025). Regrettably, most reported chitinases still exhibit low catalytic efficiency toward insoluble chitin, limiting their industrial application..In summary, the efficient discovery and engineering of novel chitinases with high activity toward natural insoluble crystalline chitin (i.e., untreated seafood shell waste) hold great significance for the efficient utilization of chitin resources. Although several reviews have summarized chitinase catalytic mechanisms and biotechnological applications (Hasan et al. 2023; Mahajan et al. 2024; Rabadiya et al. 2024). But most reviews treat individual strategies or application areas in isolation, rather than synthesizing how different approaches could be combined to address insoluble chitin. Moreover, few reviews have incorporated recent advances—such as AI-assisted enzyme design—into a unified conceptual framework. In this review, we address these gaps by proposing a multi-strategy synergy that emphasizes the coordinated integration of complementary engineering approaches to enhance chitinase performance. These include protein engineering (directed evolution and rational design), domain fusion, enzyme immobilization, AI-assisted design, and expression optimization. Each strategy primarily addresses different mechanistic or performance limitations—such as catalytic efficiency, substrate affinity, stability, and productivity—and their potential combination is expected to provide complementary benefits that may collectively enhance overall chitinase performance. This integrative framework provides a new perspective for developing next-generation chitinases for the green and efficient biotransformation of insoluble chitin and promoting the global efficient utilization of chitin resources.

## Antidegradation barriers of natural insoluble chitin

**Fig. 1:**
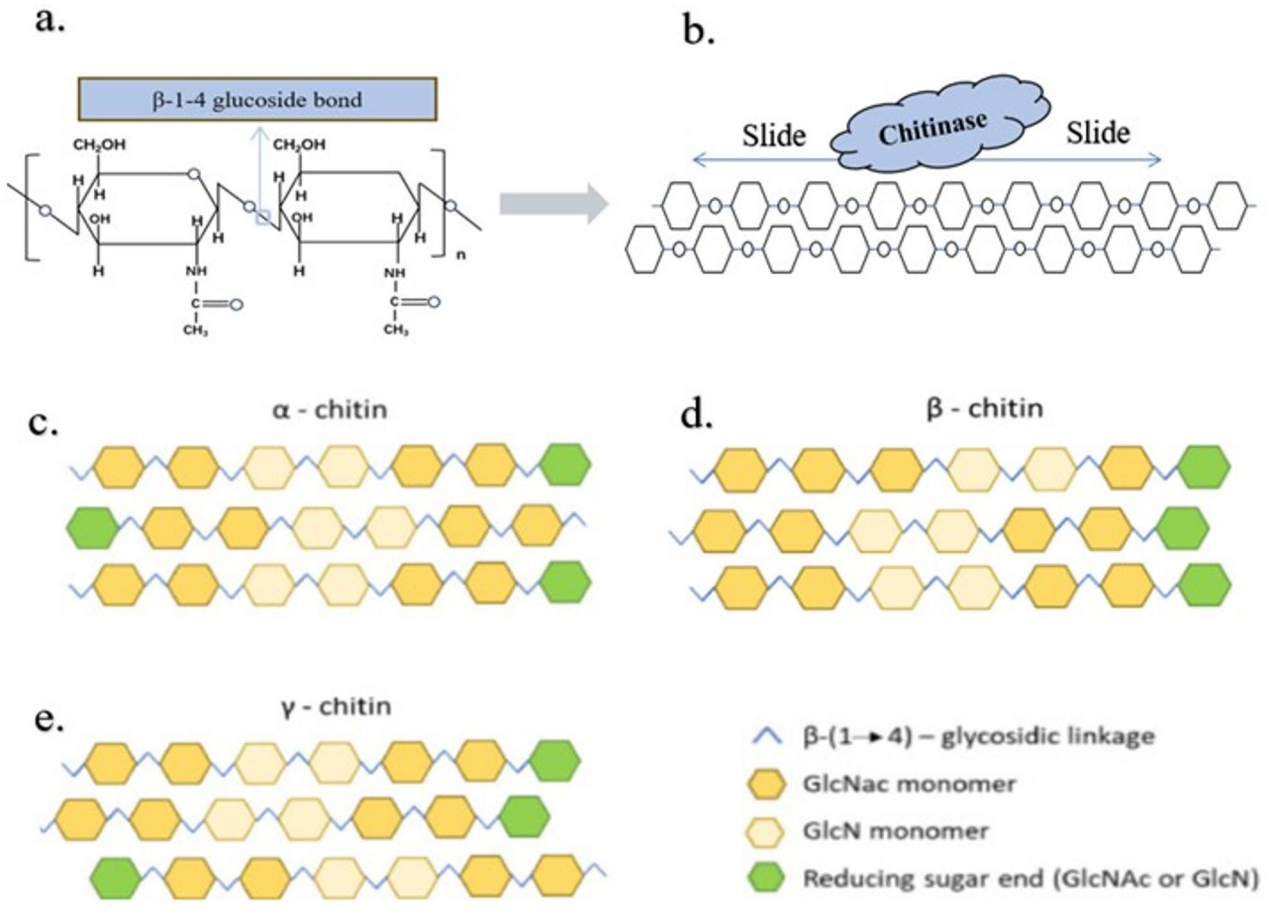
**a** Chemical structure of chitin. **b** The dense structure of chitin hinders enzyme binding to it. **c, d, e** Crystal structures of several common chitins(Chakravarty and Edwards 2022)

Chitin exists in three polymorphs (α-, β-, γ-)(Sulthan et al. 2025), all composed of N-acetyl-D-glucosamine (GlcNAc) units linked by β-1,4-glycosidic bonds. However, differences in the packing mode of molecular chains in their supramolecular structures confer distinct characteristics to each (Fig. 1). Notably, the antidegradation property of chitin originates from its unique structure: in α-chitin, adjacent molecular chains adopt an antiparallel arrangement, and extensive intermolecular hydrogen bonds form between acetamido groups (—NHCOCH₃) and hydroxyl groups (—OH) of neighboring chains(Deng et al. 2021). The extensive hydrogen-bonding network in α-chitin tightly links adjacent molecular chains, forming a highly ordered crystalline arrangement characterized by an exceptionally short interchain distance (Kadokawa 2024; Salavati 2023). Consequently, α-chitin exhibits high crystallinity, hardness, and chemical stability. It is commonly found in the exoskeletons of crustaceans (e.g. shrimp and crab shells) and fungal cell walls, making it the most abundant naturally occurring form of chitin.In contrast, all molecular chains of β-chitin are arranged in a parallel manner, and interchain associations are maintained solely by weak interactions (e.g. hydrogen bonds between hydroxyl groups)(Chakravarty and Edwards 2022). This structural feature leads to lower crystallinity and a loose architecture for β-chitin, which is typically present in the cartilage of cephalopods (e.g. squid pens). Compared with the α- and β-forms, γ-chitin exhibits intermediate intermolecular hydrogen-bonding strength and crystallinity. Structural analyses have revealed that γ-chitin adopts a mixed packing arrangement, in which two chains are aligned in an antiparallel orientation and one in a parallel configuration(Sherali 2025). This hybrid organization generates a heterogeneous hydrogen-bonding network—partially resembling the strong intersheet hydrogen bonds of α-chitin, while also containing weaker intrachain interactions characteristic of β-chitin. Consequently, γ-chitin displays moderate crystallinity and lattice spacing, showing lower swelling capacity than β-chitin but higher accessibility than α-chitin(Fernando et al. 2021; Selvoski et al. 2025). Due to its limited natural occurrence—mainly in insect cuticles, silkworm cocoons, and certain fungal cell walls—its molecular organization and enzymatic degradation mechanisms remain relatively underexplored(Shurov et al. 2022).

Regardless of the polymorph (α-, β-, or γ-), the ordered structure and strong intramolecular/intermolecular interactions of chitin restrict its solubility, thereby reducing its applicability in industrial processes. Owing to the spatial arrangement of 2,3-trans substituents in the monosaccharide unit, chitin exhibits high stability against most chemical reagents. It is insoluble in water or common solvents, irrespective of its polymorphic form(Pohling et al. 2022). Furthermore, in its natural state (e.g. crustacean exoskeletons or fungal cell walls), chitin often forms complex composites with proteins, calcium salts, and other polysaccharides. For instance, approximately 70% of proteins in squid cartilage are tightly cross-linked with chitin to form glycoprotein polymers, which severely hinders the access of enzyme molecules to the chitin substrate(Karthick et al. 2024). Both Wide-Angle X-ray Scattering (WAXS) and Small-Angle Neutron Scattering (SANS) have confirmed that the crystallinity of natural chitin can reach 60–85%(Navarro 2022), and the repeating units on the crystal surface are highly uniform, with a lack of defect sites such as chain ends or twisted regions.

## Degradation strategies for insoluble chitin

**Fig. 2:**
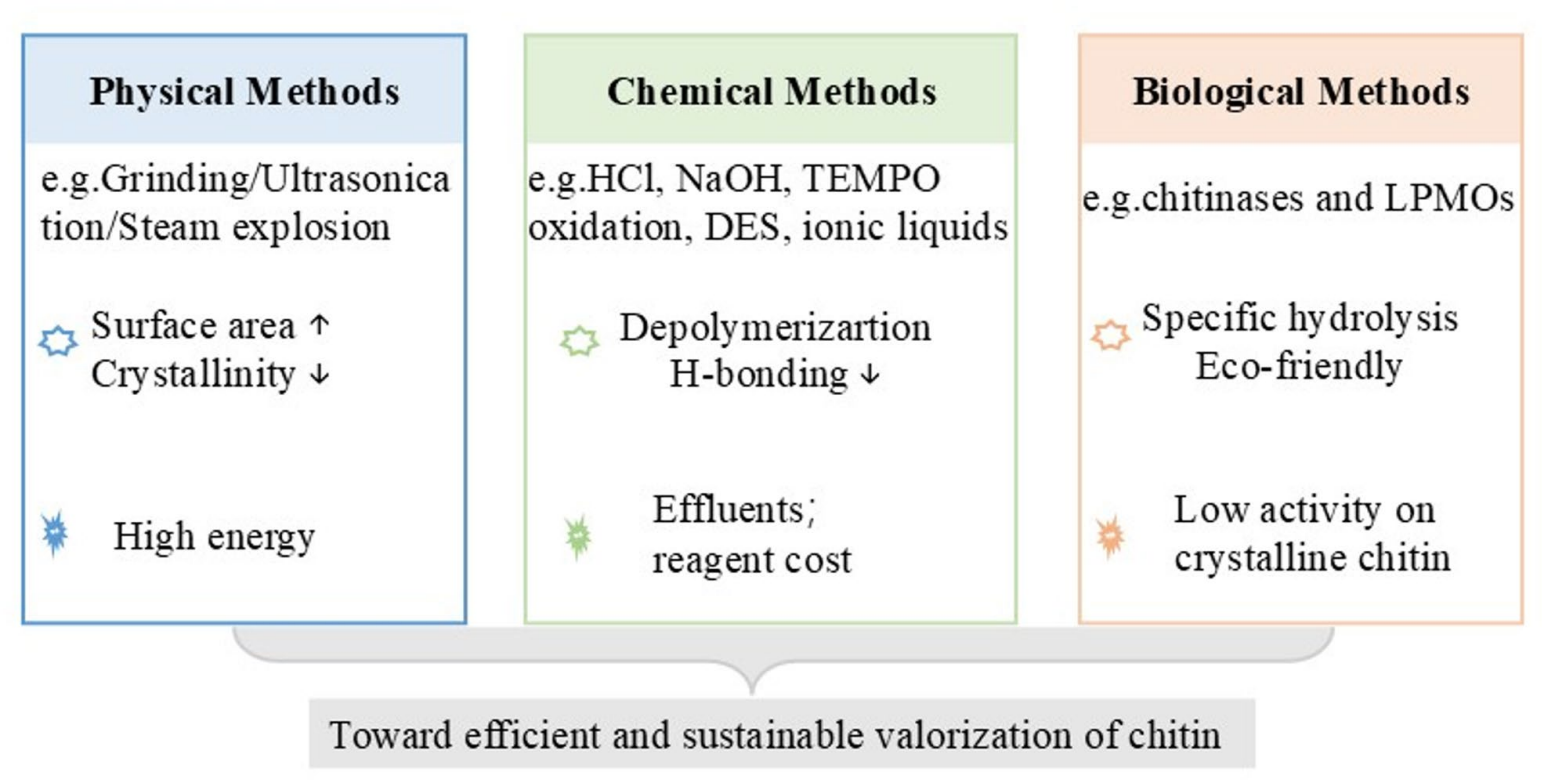
A brief introduction to the main degradation strategies for insoluble crystalline chitin

The dense hydrogen-bonding network and high crystallinity of natural chitin make it inherently resistant to chemical and enzymatic hydrolysis. To overcome these physicochemical barriers, various strategies—physical, chemical, and biological(Fig. 2)—have been developed to enhance the accessibility of insoluble crystalline chitin. These approaches differ in their mechanisms, efficiency, environmental compatibility, and scalability, as outlined below.

### Physical pretreatment approaches

Physical pretreatment methods aim to disrupt the dense hydrogen-bonding network and crystalline regions of chitin, thereby reducing particle size and increasing surface accessibility (Chokradjaroen et al. 2021). Mechanical grinding effectively breaks the macrostructure of crustacean shells but consumes large amounts of energy and yields heterogeneous particles (Kitagawa et al. 2025). Advanced techniques such as steam explosion, ultrasonication, and high-pressure homogenization further weaken intermolecular hydrogen bonds and transform α-chitin into more amorphous structures. Zhai et al. (2020) reported that steam treatment reduced crystallinity by 24.7% and increased reducing sugar yield by 40%. Similarly, Liu et al. (2022) treated shrimp shell chitin with ultra-micro grinding (UMG) combined with high-pressure homogenization (HPH); the treated chitin exhibited reduced crystallinity, increased specific surface area, and formed a reticular porous structure, with the maximum reducing sugar yield increased by 6.05 mg/mL after enzymatic hydrolysis. While Dotto et al. (2016) observed that ultrasound decreased crystallinity from 86.3% to 70.0%. Wang et al. (2020a, b) also found that ultrasound-pretreated chitin produced fourfold higher GlcNAc levels during enzymatic hydrolysis. Emerging methods such as pulsed electric field (PEF) (Psarianos et al. 2022) and supercritical CO₂ treatment (Dotto et al. 2015) can enhance surface porosity without producing harmful by-products.

Despite these advantages, physical approaches alone generally fail to fully degrade chitin and remain limited by high energy demand, low efficiency, and poor scalability. Consequently, they are often applied as environmentally friendly pretreatment steps to improve the effectiveness of subsequent chemical or enzymatic processes.

### Chemical modification strategies

Most reported studies on chitin degradation employ colloidal chitin—a partially solubilized form derived from chemically pretreated chitin—as the substrate, since this conversion markedly enhances chitinase activity (Zhao et al. 2023a, b; Xie et al. 2021; Dahiya et al. 2022). The preparation of colloidal chitin, however, is resource-intensive and environmentally burdensome. It typically begins with mechanical grinding of raw materials (e.g. crab or shrimp shells) to reduce particle size, followed by acid dissolution using concentrated HCl or similar reagents (Ngasotter et al. 2022). The resulting chitin is then precipitated and subjected to extensive washing to neutralize residual acid (Xu et al. 2024; Matroodi et al. 2024), consuming large amounts of water and producing acidic wastewater that increases treatment costs (Zhang et al. 2021a, b; Pan et al. 2019). Subsequent centrifugation or filtration adds further energy input and equipment wear (Du et al. 2021; Zhang et al. 2023). As a result, despite its advantages for enzymatic assays, colloidal chitin preparation remains costly and unsustainable for large-scale applications. Beyond colloidal chitin production, chemical modification approaches aim to weaken the hydrogen-bond network and crystalline packing of natural chitin via acid, alkali, or oxidation reactions. Acid hydrolysis using HCl or H₂SO₄ effectively depolymerizes chitin into chitooligosaccharides, while alkaline deacetylation (NaOH) converts chitin to chitosan, reducing crystallinity and enhancing solubility(Li et al. 2021; Rasweefali et al. 2025). However, both methods require high reagent input and generate corrosive effluents. Greener alternatives such as TEMPO-mediated oxidation, deep eutectic solvents (DES)(Wang et al. 2024a, b, c; Li et al. 2024), and ionic liquids can modify chitin under milder conditions and are more sustainable, though challenges remain in reagent recovery and cost.

In summary, while chemical methods efficiently disrupt chitin’s crystalline framework and facilitate substrate accessibility, they often compromise environmental safety and economic feasibility. Hence, the development of biocatalytic approaches that enable direct enzymatic degradation of natural insoluble chitin has become a key research direction for achieving green and sustainable chitin valorization.

### Biocatalytic and enzymatic strategies

Biological degradation offers the most sustainable and selective route for converting chitin into valuable bio-based products. Unlike physical and chemical pretreatments—which often require high energy input, harsh conditions, or strong chemical reagents—enzymatic hydrolysis proceeds under mild, aqueous, and environmentally benign conditions, minimizing secondary pollution while preserving the structural integrity and functionality of products (Farokhi et al. 2024). This approach primarily relies on chitinases and auxiliary enzymes such as lytic polysaccharide monooxygenases (LPMOs) (Zhao et al. 2024), which cooperatively cleave β-1,4-glycosidic bonds with high substrate specificity and regioselectivity, enabling the controlled production of chitooligosaccharides (COS) and N-acetylglucosamine (GlcNAc). These features give the biological method a clear advantage in precision, sustainability, and process compatibility compared with conventional pretreatments.

However, unlike colloidal chitin, natural crystalline chitin shows extremely low solubility and dense hydrogen-bonding networks that restrict enzymatic access and activity. AFM measurements indicate that the chitinase–substrate binding force (~ 71 pN) is insufficient to maintain a stable complex (Hou et al. 2020), while molecular simulations reveal a high interaction energy (~ =  − 12.5 kcal·mol⁻^1^) caused by acetyl-mediated hydrogen bonds (Hudek et al. 2023), collectively reflecting the strong structural resistance of α-chitin to enzymatic degradation. Furthermore, cooperative hydrogen bonding and hydration-mediated interfaces within the crystalline matrix further stabilize α-chitin and limit enzyme mobility (Hudek et al. 2023; Yurtsever et al. 2022; Qu et al. 2020).

Despite these barriers, enzymatic degradation remains the most promising and sustainable approach for chitin valorization. Advances in enzyme engineering and AI-assisted design continue to improve catalytic efficiency and substrate compatibility. The following section outlines the structural features and catalytic mechanisms of chitinases, providing a foundation for understanding how these challenges can be overcome.

## Structural features and catalytic mechanisms of chitinases

### Overall description of the chitinase family

The complex crystalline structure of insoluble chitin—such as the high-density hydrogen-bond network and interlayer cooperative stability of α-chitin—has driven the evolution of a key class of hydrolytic enzymes: chitinases. As eco-friendly catalysts specifically hydrolyzing β-1,4-glycosidic bonds, their functional diversity stems from the glycoside hydrolase (GH) families classified in the CAZY database (http://www.cazy.org). Chitinases are primarily grouped into GH18 and GH19 families based on sequence similarity(Zhang et al. 2025; Poria et al. 2021). These enzymes typically consist of multiple domains: a core catalytic domain flanked by substrate recognition/binding domains such as carbohydrate-binding modules (CBMs) or fibronectin type III (Fn Ⅲ) domains(Honda et al. 2019). Chitin-binding domains (ChBDs), commonly found in chitinases(Xue et al. 2021; Eichfeld et al. 2025), belong mainly to the CBM5, CBM12, and CBM18 families. This modular architecture forms the basis for specific degradation, with domains acting synergistically to achieve precise chitin recognition and cleavage.

### GH18 chitinases: structure and mechanism

**Fig. 3:**
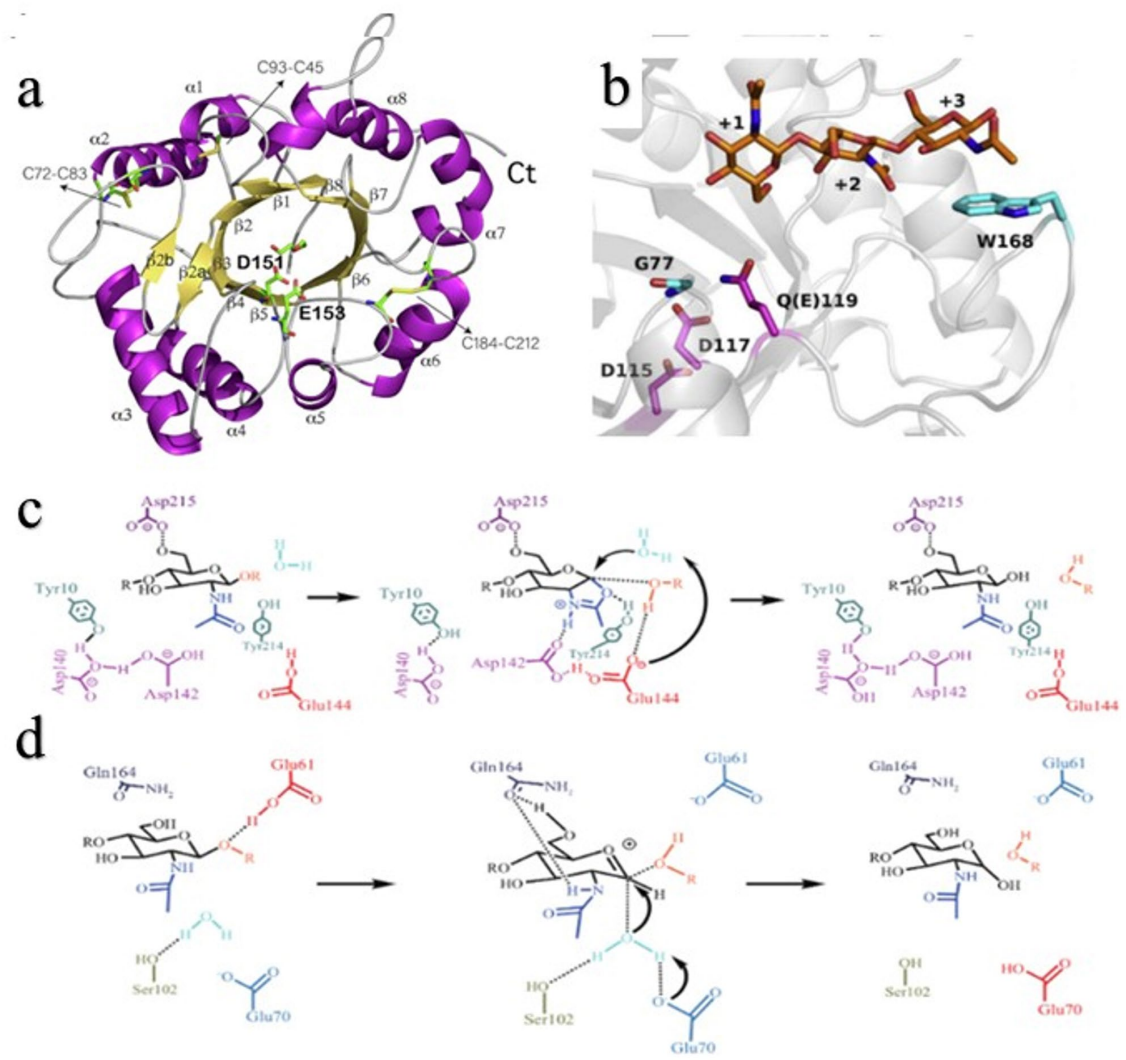
Schematic diagram of the characteristic structures and degradation mechanisms of chitinases. **a** Canonical (β/α)_8_ TIM barrel structure of chitinases(Minguet et al. 2025). **b** Aromatic amino acids around the active pocket guide the substrate to approach the active site via π-π stacking interactions(Nagata et al. 2021). **c, d** Degradation mechanisms of chitinases from the GH18 and GH19 families(Zhao et al. 2022)

GH18 chitinases, the most diverse family (sources: fungi, bacteria, plants, animals), feature a canonical (β/α)_8_ TIM barrel catalytic domain(Sun et al. 2019), composed of 8 α-helices and 8 β-sheets connected by flexible loops (Fig. 3a)(Minguet et al. 2025; Madhuprakash et al. 2025). The catalytic cleft, located above the barrel, contains conserved DxxDxDxE catalytic modules and SXGG substrate-binding modules(Guo et al. 2024). GH18 enzymes possess a long, deep substrate-binding cleft with multiple subsites, where conserved aromatic amino acid residues at the active center surface bind chitin through hydrophobic and π–π stacking interactions(Fig. 3b)(Nagata et al. 2021; Umemoto et al. 2015). Recent high-resolution structural and computational analyses (Niedzialek et al. 2025) have revealed that the catalytic efficiency of GH18 enzymes relies heavily on loop dynamics. A flexible tryptophan-containing loop acts as a “tryptophan lid” to regulate substrate entry and product release, while a conserved tyrosine functions as a “molecular piston,” transmitting mechanical energy that stabilizes the oxazolinium-ion intermediate and enhances transition-state formation. These motions couple to the substrate-assisted retaining mechanism, in which the 2-acetamido group of GlcNAc acts as a nucleophile (Van et al. 2001; Zhao et al. 2022; Minguet et al. 2024). For instance, in *Serratia marcescens* chitinase SmChiB (PDB ID: 1E6N), aromatic residues anchor the substrate hydrophobically. When GlcNAc occupies the − 1 subsite, the enzyme induces a chair-to-boat conformational shift, enabling nucleophilic attack at C1. The oxazolinium intermediate then dissociates as Glu144 resets and Asp142 reorients (assisted by Asp140), completing hydrolysis (Fig. 3c). Further mechanistic refinement from recent cryo-EM and QM/MM simulations demonstrates a dynamic catalytic pathway: the β/α loops undergo transient closure during substrate binding, lowering the transition-state barrier. Additionally, dual-site cooperativity between catalytic residues—especially the secondary Asp or Glu acting as a proton-transfer bridge—stabilizes the oxazolinium intermediate. A water-mediated hydrogen-bond network within the active pocket dynamically tunes catalytic geometry, maintaining an ideal nucleophile–substrate distance. Meanwhile, electrostatic potential focusing by flanking Arg and Lys residues polarizes the glycosidic C1–O bond, facilitating cleavage(Minguet et al. 2025). Together, these multiscale effects illustrate that GH18 catalysis involves a complex interplay of active-site chemistry, conformational mobility, and electrostatic modulation.

### GH19 chitinases: structure and mechanism

GH19 chitinases are primarily distributed in plants, with rare bacterial homologs originating from horizontal gene transfer(Prakash et al. 2010; Ma et al. 2025). Structurally, they feature a compact α-helical catalytic core and several variable loops forming a shallow, open substrate-binding groove (Tanaka et al. 2017). These flexible loops extend toward the glycosyl and aglycon subsites (− 2, − 1, + 1, + 2) (Oyeleye et al. 2018), enabling dynamic adaptation to chitin chain length and orientation. Unlike GH18 chitinases, GH19 enzymes lack the (β/α)_8_ TIM-barrel and adopt an inverting single-displacement mechanism(Fig. 3d), in which two conserved glutamate residues act as proton donor and base catalyst, respectively (Orlando et al. 2021; Kawamoto et al. 2022; Irum et al. 2025). One Glu protonates the glycosidic oxygen, while the other activates a water molecule for nucleophilic attack at C1, directly inverting the anomeric configuration without forming an oxazolinium intermediate. Crystallographic and molecular dynamics studies show that aromatic residues (Trp, Tyr, Phe) lining the binding groove stabilize the substrate through CH–π and hydrogen-bond interactions (Ohnuma et al. 2013; Tanaka et al. 2017). The flexibility of terminal loops modulates groove opening and closing during catalysis, allowing efficient hydrolysis of soluble or partially amorphous chitin. Thus, GH19 chitinases complement GH18 enzymes by combining an open, flexible architecture with a water-mediated inverting catalytic route suited for plant-type chitin degradation.

### Shared features and catalytic constraints of GH18 and GH19 chitinases

Despite their distinct structural frameworks, GH18 and GH19 chitinases share several catalytic features critical for chitin hydrolysis. Both rely on aromatic and hydrophobic residues within their binding grooves to anchor GlcNAc units through π–π and CH–π interactions, while carbohydrate-binding modules (CBMs) enhance enzyme accessibility by increasing contact with insoluble chitin surfaces (Madland et al. 2019; Stavros et al. 2015). However, their overall catalytic turnover remains constrained by the intrinsic rigidity of crystalline α-chitin, where the dense hydrogen-bonding network and antiparallel chain packing hinder substrate rearrangement and enzyme penetration (Khan et al. 2017; Sivaramakrishna et al. 2020). Consequently, even structurally optimized chitinases display limited activity toward native substrates.

Recognizing these common catalytic limitations provides the mechanistic rationale for subsequent engineering strategies that aim to overcome these structural and energetic barriers. These approaches are discussed in detail in the following section.

## Enzyme protein engineering for insoluble chitin

The complex crystalline structure of insoluble chitin severely hinders the enzymatic hydrolysis process, requiring innovations in enzyme engineering technologies to break through the bottleneck of its degradation efficiency. Current research focuses on strategies such as directed evolution, rational design, modification of substrate-binding domains, and mining or designing novel enzyme resources, aiming to obtain enzymes with efficient degradation activity toward insoluble chitin and promote their practical industrial application.

### Irrational design of chitinases

Directed evolution (e.g. irrational design) is a method that simulates natural evolution. It relies on creating a large number of protein mutants with random variations, constructing these mutants into a diverse library, and then screening to select mutants with desired properties under specific pressures. Currently, commonly used directed evolution techniques include error-prone PCR, DNA shuffling, exon shuffling, random primer in vitro recombination, site-saturation mutagenesis, and staggered extension recombination(Ouyang et al. 2025). In studies on the directed evolution of chitinases, molecular biology tools and high-throughput screening technologies are also used, forming a widely applied analytical workflow. Fan et al. (2007) improved the catalytic activity of Bbchit1 from *Beauveria bassiana* via directed evolution. After three rounds of DNA shuffling, a library containing 150,000 variants (point mutation frequency of 0.6%) was constructed, and two variants with enhanced activity—SHU-1 (36% higher activity) and SHU-2 (56% higher activity)—were screened out. Among the mutation sites of these two variants, V279E in SHU-1 and D99N/A309E in SHU-2 are located outside the two identified substrate-binding sites and catalytic region of Bbchit1. These substitutions enhance enzyme activity by inducing long-range conformational regulation, where distal mutations alter inter-domain packing and loop flexibility, thereby optimizing active-site geometry and turnover efficiency. Similarly, directed evolution of chitinase from *Bacillus licheniformis* showed(Songsiriritthigul et al. 2009) that mutation sites are distributed in the catalytic domain and fibronectin type III (FnIII) domain: Val234Ala in the catalytic domain is located at the end of helix 4 of the (β/α)_8_ barrel, which may affect inter-helix interactions and thus alter the conformational flexibility of the enzyme(Nestl et al. 2014; Xie et al. 2014); Arg387Gln is located on the surface of the α + β insertion domain, which may enhance long-range interactions with substrates through charge changes(Jiang et al. 2020); mutations in the FnIII domain may indirectly improve activity by affecting its interaction with the catalytic domain. None of these sites is directly involved in catalysis, indicating that the enhancement of enzyme activity may not be achieved by directly altering key sites for catalysis or substrate binding, but rather through long-range conformational regulation.

In contrast, variants such as BcChiA1 and Chisb display features consistent with catalytic-center electronic effects. Substitutions adjacent to the catalytic residues reshape the electrostatic environment, shifting local pK_a values and improving transition-state stabilization (Wang et al. 2020a, b; Pan et al. 2019). This type of regulation highlights that even subtle electronic tuning can yield substantial kinetic improvements. Meanwhile, other enzymes—such as Chi-a—demonstrate substrate recognition/binding optimization, where mutations fine-tune binding loops or peripheral pockets to enhance productive substrate orientation, leading to improved catalytic efficiency (Selvaraj et al. 2009).

This suggests the complexity of enzyme activity regulation and highlights the advantage of directed evolution in identifying unexpected key sites(Table 1). This technology can significantly improve key enzymatic properties of chitinases, such as catalytic efficiency and enzyme activity(Chen et al. 2024a, b, c; Yu et al. 2012), but it is also prone to unintended consequences(Hibbert et al. 2005), leading to poor performance in practical applications(Martinez et al. 2012)—even the enzymatic properties of mutants are far lower than those of the wild type. Additionally, this technology requires the construction of mutant libraries and the implementation of multiple rounds of screening, resulting in high costs. However, in recent years, microfluidic chip technology(Yuan et al. 2022) can increase the screening throughput to 10⁶ per hour and realize real-time detection of enzyme activity at the single-cell level. For example, Menghiu et al. (Menghiu et al. 2021) successfully screened mutants with twice the activity of the wild type using a screening method based on fluorescence-activated cell sorting (FACS). FACS has gradually become a tool for enzyme library screening due to its high sensitivity and ability to detect up to 10⁷ enzymes per day. Despite its great potential, FACS screening is still not widely used due to limited detection compatibility.

**Table 1:** Summary of selected directed evolution studies on chitinases

Enzyme (source)	Method	Substrate	Activity change (vs WT)	Proposed mechanism	Ref
Bbchit1	Directed evolution	colloidal chitin	V279E(+ 36%) D99N/A309E(+ 56%)	Conformational Regulation	Fan et al. 2007
*Bacillus licheniformis* chitinase	Directed evolution	p-nitrophenyl-β-1,4-N,N'-diacetyl-chitobiose	k_cat_/K_m_ improved 2.7/2.3-fold	Conformational Regulation	Songsiriritthigul et al. 2009
BcChiA1	Directed evolution	CC-RBB	16.89-fold	Catalytic Center Electronic Effect	Wang et al. 2020a, b
*T. harzianum* Chit42	Directed evolution	colloidal chitin	1.26-fold	Conformational Regulation	Chai et al. 2019
Chi-a	Directed evolution	colloidal chitin	1.1-fold	Substrate Recognition /Binding Optimization	Selvaraj et al. 2009
Chisb	Directed evolution	colloidal chitin	1.57-fold	Catalytic Center Electronic Effect	Pan et al. 2019
PbChi70	Directed evolution	colloidal chitin	2.4-fold	Conformational Regulation	Han et al. 2024a, b

### Precision mutagenesis based on structural insights: optimization of substrate-specific activity and thermostability

Rational design is an enzyme engineering technology distinct from directed evolution. The former relies more heavily on structural and functional information of enzymes during design, with clear objectives. The basic workflow of this technology is as follows: first, three-dimensional structural information of the enzyme is obtained using X-ray crystallography, nuclear magnetic resonance (NMR)(Song et al. 2023), or other high-resolution structural analysis techniques. Based on this structural information, key residues involved in substrate binding and the catalytic mechanism are identified, and the structural characteristics of the active site are clarified. Then, through computer simulation, a large number of compounds or mutants are screened(Pongsupasa et al. 2021) to predict their binding ability to the enzyme and their impact on enzyme activity. Based on the above analysis, specific amino acid mutations are designed to enhance the enzyme’s stability and activity, or to alter its substrate specificity(Yan et al. 2022). Finally, experiments are conducted to verify the results of rational design and evaluate the actual performance of the designed products.

**Fig. 4:**
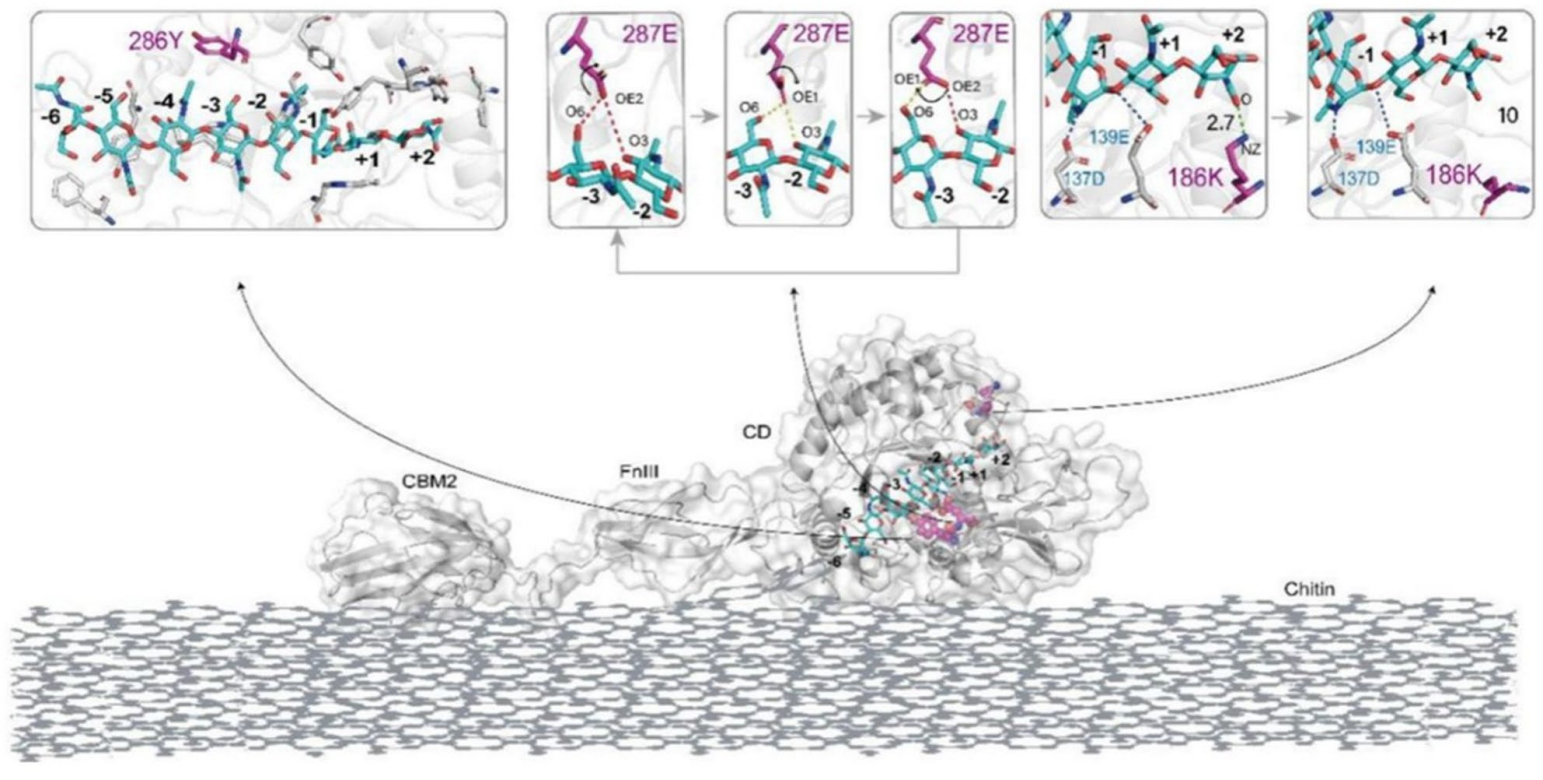
The role of three residues in the ratchet model of processive catalysis in SsChi18A(Zhao et al. 2023a, b)

Site-directed mutagenesis is a commonly used technique in rational design, which involves precise substitution of key amino acid sites in the chitinase gene sequence to alter its spatial structure or catalytic active center, thereby enhancing enzyme activity(Table 2). In recent years, research reports on this technology have continued to increase, among which the precise regulation of enzyme activity through site-directed mutagenesis of key residues has become a core direction to break the bottleneck of insoluble chitin degradation. The study by Zhao et al. (2023a, b) on the thermophilic chitinase SsChi18A from *Streptomyces* is highly representative: by analyzing the enzyme’s modular structure and key residues (Fig. 4), it was found that the carbohydrate-binding module CBM2 anchors chitin crystal substrates, the fibronectin type III domain FnIII maintains conformational stability(Sidar et al. 2020), and the catalytic domain (CD) serves as the catalytic core—its deep cleft contains subsites from -6 to + 2, which can accommodate chitin chains to complete the binding and hydrolysis process. Many aromatic residues (e.g. Y286) stabilize chitin chains in the catalytic domain cleft via CH-π interactions(Spiwok et al. 2017), ensuring substrate positioning. Polar residues (e.g. E287) form alternating hydrogen bonds between their side-chain carboxyl groups and the O3/O6 atoms of the substrate, driving chitin chains to slide along the cleft(Hamre et al. 2015) and providing momentum for the enzyme to continuously degrade crystalline substrates. Flexible residue K186 is located at the product release end: in the early stage of catalysis, short hydrogen bonds "capture" products to prevent escape; after degradation is completed, the hydrogen bonds break to release products, ensuring continuous degradation. Site-directed modification of these key active sites resulted in the activities of variants Y286W, E287A, and K186A toward colloidal chitin increasing to 151%, 135%, and 129% of that of the wild type, while the activities of F48W and the double mutants F48W/Y209F and F48W/Y286W toward crystalline chitin increased by 35%, 16%, and 36%, respectively. Notably, however, the activities of Y286W, E287A, and K186A toward crystalline chitin decreased instead. The reason for this contradictory phenomenon lies in the fact that colloidal chitin is a soluble substrate, and the enzyme’s degradation efficiency depends more on catalytic turnover rate rather than sustained binding capacity. After the mutation of Y286 to Trp, the indole ring of Trp expands the hydrophobic interaction space between the enzyme and the substrate, which optimizes the turnover rate of colloidal substrates; however, the overly strong hydrophobic environment in the substrate-binding cleft "traps" chitin chains, making it difficult for them to slide, resulting in reduced processivity and thus decreased activity toward crystalline chitin. E287 originally drives chitin chains to slide along the cleft, but such "forced sliding" is unnecessary for colloidal chitin(Jiménez et al. 2021; Nakamura et al. 2018); after mutation to Ala, the absence of a polar side chain reduces electrostatic interference between the enzyme and the substrate, making the catalytic center more flexible and better adapted to the flexible structure of colloidal chitin. At the same time, Ala cannot form such a dynamic pulling force, so the enzyme cannot continuously degrade crystalline chitin, leading to decreased activity. K186 controls product release, but products of colloidal chitin are more prone to diffusion; after mutation to Ala, the simplified side chain of Ala eliminates restrictions on product release, resulting in a significant increase in kcat when using colloidal chitin as the substrate, while its processivity collapses, leading to reduced activity toward crystalline chitin.

**Table 2:** Some Reported Chitinase Mutants with Improved Catalytic Performance

Enzyme (source)	Mutation / Modification	Substrate	Activity/ Thermostability (vs WT)	Ref
SsChi18A	Y286W E287A K186A F48W	colloidal chitin colloidal chitin colloidal chitin crystalline chitin	169.7 IU/mg(+ 51%) 151.8 IU/mg(+ 35%) 144.8 IU/mg(+ 29%) 4.87 IU/mg (+ 35%)	Zhao et al. 2023a, b
Serratia marcescens B4A	G191V S390I	colloidal chitin	Withstood up to 90 °C; 15-fold longer half-life at 60 °C Stable after 90 min at 70 °C	Emruzi et al. 2018
*Chi304* (Thermophilic GH18)	F79A, M264L, W140R, W272R (single) F79A/W140R M264L/W272R (double)	(GlcNAc)₆ / Colloidal chitin (GlcNAc)₆	Endo-activity improved 1.24–1.89-fold Endo-activity improved 1.67–2.06fold	Guan et al. 2024
PoChi	Y240W F268W	colloidal chitin crystalline chitin	Improved ~ 1.2 fold Improved ~ 1.1 fold	Chen et al. 2024a, b, c
Chi18A Chi18C Chi18D	E315A E315A E408A	colloidal chitin	Activity lost completely	Kim et al. 2021
BcChiA1	N257Y/N271E/K177R/S67G/A220V/A279V(five)	colloidal chitin	half-life (from 5 to 295 min) at 60°C	Liu et al. 2025
Chi1	D615S S242A-D615S (double)	colloidal chitin	t₁/₂(45 °C):2.5 → 60 h (+ 24 ×); residual activity 51.4% after 72 h t₁/₂(45 °C):38 h(15 × WT	Zhou et al. 2025
SsChi18A	Q41K Q41R A189V	colloidal chitin	activity + 23; t₁/₂ + 6 h activity + 30%; t₁/₂ + 7.5 h activity + 34%; t₁/₂ + 35 h	Zhao et al. 2025

Therefore, in rational design, one should not be limited solely to the role of residues in traditional enzymatic hydrolysis mechanisms, but rather comprehensively consider the characteristics of interactions between the enzyme and different substrates. For example, for crystalline chitin, it is necessary to strengthen the function of processive residues(Qu et al. 2020), while for colloidal chitin, focus should be placed on residues related to product release. In addition, the multifaceted impact of mutations on the overall performance of the enzyme must be considered. Through precise mutagenesis strategies, effective enhancement of chitinase activity can be achieved, providing strong support for the efficient degradation of insoluble chitin.

Beyond modifying activity, enhancing the thermostability of chitinases is crucial for improving their industrial application potential. Emruzi et al. replaced glycine at position 191 with valine in the wild-type chitinase(Emruzi et al. 2018). After the G191V mutation, the mutant exhibited similar pH stability to the wild-type chitinase; however, structural analysis revealed that this mutation shortened the key loop, extended the adjacent β-sheet, reduced the flexibility of the unfolded state, and enhanced structural rigidity. Through this entropic stabilization mechanism, thermostability was significantly improved: the mutant chitinase could withstand temperatures up to 90°C for 90 min, and its half-life at 60°C was 15 times longer than that of the wild-type. In *Paenibacillus pasadenensis* CS0611, chitinase stability was enhanced via semi-rational design and the introduction of disulfide bonds, resulting in a variant with a 26.3-fold longer half-life at 50°C(Xu et al. 2020). Although mutations in surface flexible loops and the introduction of disulfide bonds have effectively improved chitinase thermostability, more comprehensive strategies are still required to further optimize the enzyme’s performance in industrial scenarios.

Loop dynamics have emerged as a critical determinant of enzyme performance. Zhao et al. (2025) demonstrated that the flexible loop2 and loop4 of GH18 chitinase SsChi18A fine-tune the balance between activity and stability. Mutations A43T and Q41R stabilized loop2 via enhanced hydrogen bonding, extending enzyme half-life by up to 7.5 h and increasing activity by 30%. Similarly, A189V strengthened loop4 interactions with the catalytic Glu, improving hydrophobic packing and prolonging thermal stability by 35 h. These results highlight a function-driven strategy based on *loop dynamics perturbation*, which effectively overcomes the traditional activity–stability trade-off, providing a framework for thermostable enzyme design.

However, site-directed mutagenesis alone may cause trade-offs, such as decreased pH adaptability or flexibility (Ni et al. 2015). Therefore, future rational design should adopt a multi-parametric optimization framework integrating structural rigidity, loop dynamics, and substrate-dependent catalysis. For crystalline chitin, strengthening processive and binding residues (Qu et al. 2020) is beneficial, whereas for colloidal chitin, optimizing product-release residues and flexible loops is more effective. Through such precision-guided strategies, rational and semi-rational design together are driving the development of next-generation chitinases with enhanced stability, activity, and industrial potential.

### Substrate-binding domain engineering: enhancing binding affinity and catalytic activity

In research aimed at specifically enhancing the catalytic activity of chitinases toward insoluble chitin, substrate-binding domain engineering emerges as a pivotal strategy. This approach seeks to optimize enzyme performance through the precise modulation of interaction modes between chitinases and their substrates. An analysis of the aforementioned molecular mechanisms reveals that, for insoluble substrates such as chitin, the substrate-binding domain assumes unparalleled importance, given that effective binding between the enzyme and insoluble substrate constitutes a prerequisite for the exertion of degradative function(Table 3).

**Table 3:** Some Substrate-Binding Domain Engineering of Chitinases and Its Effects on Catalytic Activity

Enzyme (source)	Method	Substrate	Specific activity change (vs WT)	Proposed mechanism	Ref
ChitinaseA	binding domain tryptophan residues engineering	Colloidal chitin	Decreased from 12.6 U/mg to 2–3 U/mg (− 75–85%)	Aromatic residues mediate CH–π interactions critical for substrate binding and chain guidance	Uchiyama et al. 2001
PoChi	binding domain tryptophan residues engineering	Crystalline chitin	Decreased from 8.5 U/mg to 1.2–1.5 U/mg (− 82–95%)	Aromatic residues essential for hydrophobic π–π stacking with crystalline chitin	Chen et al. 2024a, b, c
PoChi-FnIII-ChBD PoChi-ChBD ChBD-PoChi	C-terminal fusion of FnIII–ChBD tandem domain C-terminal ChBD fusion N-terminal ChBD fusion	Crystalline chitin Crystalline chitin Crystalline chitin	15.4 U/mg (+ 83%) vs WT (8.4 U/mg) 12.7 U/mg (+ 51%) vs WT (8.4 U/mg) 11.3 U/mg (+ 34%) vs WT (8.4 U/mg)	Enhanced substrate adsorption via electrostatic and hydrophobic complementarity C-terminal ChBD reduces steric hindrance and facilitates substrate access N-terminal ChBD may sterically hinder the catalytic cleft	Chen et al. 2024a, b, c Chen et al. 2024a, b, c Chen et al. 2024a, b, c
CdChi	N-terminal fusion of *Pyrococcus furiosus* ChBD	Crystalline chitin	Increased from 2.1 U/mg to 10.5 U/mg (+ 400%); Kd decreased 13.9 → 6.3 µg	Three Trp residues enhance hydrophobic binding and substrate adsorption	Sun et al. 2023
BliGH–CeBD	Deletion of native ChBD and fusion of cellulose-binding domain (CeBD) at C-terminus	Crystalline chitin	3.8 U/mg, ~ 2 × higher than ΔChBD mutant (1.9 U/mg)	CeBD provides a polysaccharide-binding surface, enhancing substrate affinity	Neeraja et al. 2010
Chit46–ChBD	C-terminal ChBD fusion	Crystalline chitin	Increased from 4.7 U/mg to 15.0 U/mg (+ 219%)	ChBD improves adsorption and catalytic efficiency on insoluble substrate	Gu et al. 2025
Chit33–CBD	C-terminal fusion of cellulose-binding domain (CBD) via CBHII linker	Crystalline chitin	10.2 U/mg(α)vs WT 5.1 U/mg; COS yield + 30–85%	CBD (CBM1) promotes substrate delivery and increases COS product diversity	Martínez et al. 2025
R-SaChiA4	Modification of ChBDs (three variants)	Colloidal chitin	98.5 U/mg (+ 49%) vs WT (66.1 U/mg); slightly reduced thermostability	ChBD redesign enhances binding affinity but compromises thermal stability	Su et al. 2021

Suginta et al. (2016) have validated the distinct physiological role of the chitin-binding domain of chitinase A from marine bacteria in chitin-chitinase interactions. Further investigations have demonstrated that specific aromatic residues on the surface of the binding domain engage in substrate-specific interactions with chitin(Uni et al. 2012; Zakariassen et al. 2009). For instance, in chitinase A from *Serratia marcescens*, when tryptophan residues (Trp-69, Trp-33, or Trp-245) on the surface of its binding domain are substituted with alanine, the enzyme exhibits a marked reduction in binding activity toward highly crystalline β-chitin and colloidal chitin. Conversely, phenylalanine-232 (Phe-232) plays a critical role in guiding chitin chains into the catalytic cleft(Uchiyama et al. 2001).In a study conducted by Chen et al. (Chen et al. 2024a, b, c), mutation of aromatic residues (e.g. Trp-87, Trp-246) within the substrate-binding domain of wild-type PoChi resulted in an 82%–95% decrease in activity toward insoluble chitin—reaffirming the core role of aromatic residues in the binding of crystalline substrates. Furthermore, direct engineering of substrate-binding domains has been accomplished by fusing the chitin-binding domain and fibronectin domain to both termini of chitinases via three fusion strategies, which substantially improved enzymatic activity. Among the engineered constructs, the fused chitinase PoChi-FnIII-ChBD exhibited the optimal performance, with an activity of 15.4 U/mg toward insoluble chitin—representing an 83% enhancement relative to the wild-type enzyme. In contrast, the N-terminally fused variant (ChBD-PoChi) showed a 34% increase in activity, which was lower than the 51% enhancement observed for the C-terminally fused variant (PoChi-ChBD)(Chen et al. 2024a, b, c). This phenomenon may be attributed to the fact that the N-terminal ChBD forms steric hindrance with the catalytic domain of PoChi, thereby impeding substrate access to the active center(Tian et al. 2023). By comparison, the C-terminally fused ChBD, being distant from the catalytic domain, enables unimpeded binding to the substrate surface. Moreover, the incorporation of ChBD markedly improves the enzyme’s adsorption capacity toward insoluble chitin: the binding rates of PoChi-ChBD and PoChi-FnIII-ChBD were 66.7% and 72.3% higher than that of the wild-type enzyme, respectively. This improvement is likely driven by electrostatic complementarity between the positive charges of ChBD and the negative charges on the chitin surface, coupled with strengthened hydrophobic binding between tryptophan residues and chitin sugar rings via CH-π interactions(Deng et al. 2020). Similarly, fusion of the hyperthermophilic ChBD from *Pyrococcus furiosus* (containing three conserved Trp residues that enhance hydrophobic binding) to the N-terminus of Cdchi resulted in a 400% increase in catalytic activity relative to ActChi. Concurrently, a significant improvement in crystalline chitin-binding capacity was observed, with a dissociation constant (Kd) of 6.3 μg—substantially lower than the 13.9 μg measured for ActChi(Sun et al. 2023). Analogously, Neeraja et al. (Neeraja et al. 2010) developed two mutants (BliGH and BliGH-CeBD) via polymerase chain reaction (PCR)-mediated deletion of the chitin-binding domain from the chitinase of *Bacillus licheniformis*, followed by fusion of a cellulose-binding domain (CeBD) to the enzyme’s C-terminus. Relative to the deletion mutant, the fused variant (BliGH-CeBD) exhibited higher affinity toward chitin. Additionally, fusion of the ChBD from *Bacillus subtilis* to the C-terminus of chitinase Chit46 led to a 219% increase in the chimeric enzyme’s activity toward insoluble substrates(Gu et al. 2025). Su et al. (Su et al. 2021) generated three variants of chitinase SaChiA4 through modification of its chitin-binding domains (ChBDs). Among these variants, R-SaChiA4 displayed a specific catalytic activity of 98.5 U/mg toward colloidal chitin—nearly 49% higher than that of wild-type SaChiA4—though a reduction in thermostability was observed for this variant.

**Fig. 5:**
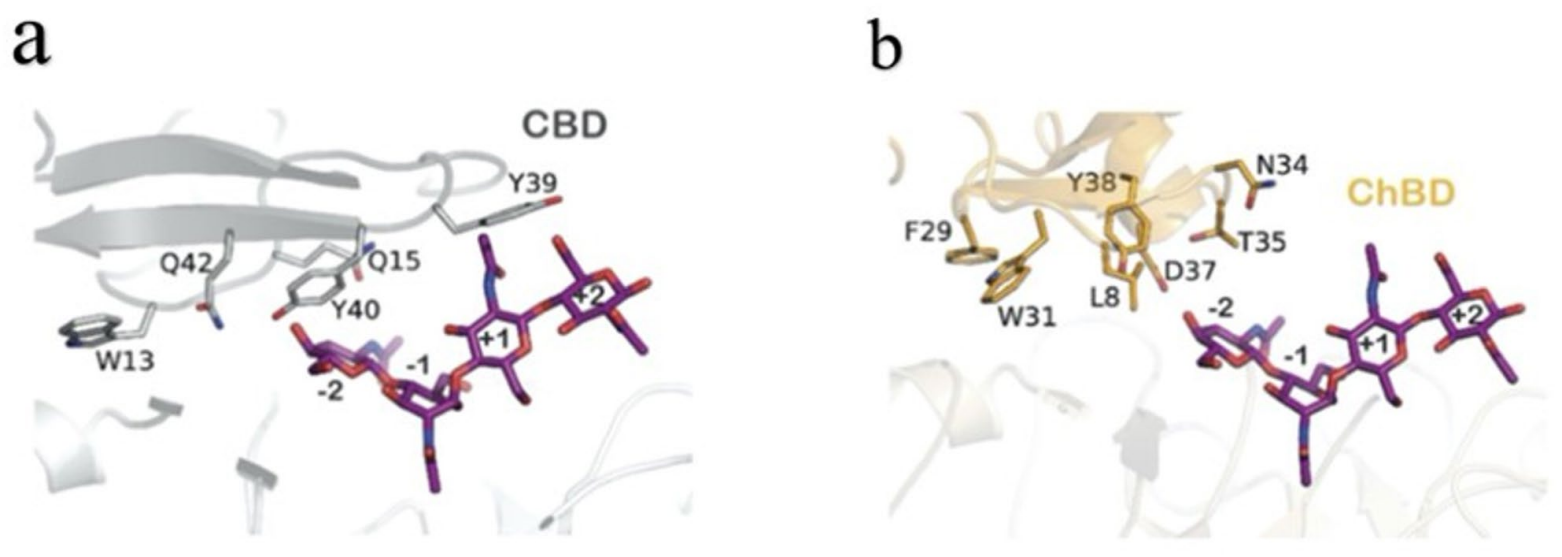
Overall structures and substrate-binding-related residues of Chit33-CBD and Chit33-ChBD(Martínez et al. 2025)

Domain engineering exhibits broad application potential in enhancing catalytic activity, binding affinity, and other key enzymatic properties. Factors, including the orientation of domain fusion and the selection of suitable domains, modulate the overall catalytic function of the protein(Chen et al. 2024a, b, c). Martínez-Ranz et al. demonstrated in their study that the choice of binding domain is critical for regulating enzymatic activity and product specificity(Martínez et al. 2025). In this work, domain engineering was conducted on the endochitinase Chit33 from *Trichoderma harzianum*, and chimeric enzymes were generated via the fusion of a cellulose-binding domain (CBD) and a chitin-binding domain (ChBD). Results indicated that the CBD substantially enhanced the enzyme’s activity toward both colloidal and crystalline chitin (with activity toward α-chitin doubled), markedly increased the yield of chitooligosaccharides (COS)—achieving a 30% increase for α-chitin-derived COS and an 85% increase for β-chitin-derived COS—and expanded product diversity to encompass 1–9 N-acetylglucosamine units. In contrast, although the ChBD conferred higher binding affinity, it reduced enzymatic activity, with tetra-N-acetylchitotetraose (NAG₄) identified as the major product. Structural analysis of these two domains revealed that the CBD is classified into the CBM1 family, which possesses a flat binding surface enriched in aromatic residues (Fig. 5). This surface enables binding to the surface of crystalline chitin via π-π stacking interactions(Tokunaga et al. 2020). Furthermore, the relative orientation between the CBD and the catalytic domain promotes the directional delivery of substrates to the active center (e.g. residues W13 and Y39 assist in substrate positioning). By comparison, the ChBD belongs to the CBM18 family; its excessively high binding strength may lead to the “trapping” of the enzyme on the substrate surface(Halling et al. 2005; Tjoelker et al. 2000), thereby reducing turnover efficiency and ultimately resulting in decreased activity. During the modification of binding domains—particularly in the fusion of heterologous domains—linker sequences are critical for preserving the overall conformational stability of the enzyme and ensuring the normal execution of its catalytic function(Kawashima et al. 2016). These linkers are primarily composed of amino acids such as glycine (Gly), serine (Ser), and proline (Pro). Glycine, whose side chain consists solely of a hydrogen atom, confers high flexibility, allowing domains to rotate freely for orientation adjustment. The hydroxyl group of Ser can stabilize local conformations via hydrogen bonding(Reddy et al. 2013, Ceballos et al. 2021), while Pro introduces a certain degree of rigidity through its cyclic structure—preventing disordered collisions between domains caused by excessive flexibility(Chen et al. 2013). For instance, the CBHII linker employed in the fusion of Chit33 with CBD/ChBD(Datta et al. 2013) is enriched in Ser and Pro; AlphaFold predictions indicate that this linker constitutes a highly disordered region. This flexibility ensures that the CBD/ChBD and the catalytic domain can dynamically adjust their relative positions in response to substrate binding requirements. Thus, in practical enzyme engineering, targeted design of domains and linker sequences should be implemented through the integration of substrate characteristics (e.g. crystallinity, solubility), functional requirements (e.g. catalytic activity, product specificity), and application scenarios (e.g. reaction temperature, reusability) to achieve maximal improvement in enzyme performance.

### Enzyme immobilization on carriers for enhanced activity and thermostability

As a core pillar in the field of biotechnology, enzyme immobilization technology restricts the free diffusion ability of naturally free enzymes by either confining them within a specific spatial range or attaching them to the surface of solid carriers. Owing to its high efficiency and operational simplicity, this technology has been widely applied—it not only significantly enhances the catalytic efficiency and operational stability of enzymes but also alters the enzyme’s substrate recognition preference and enantioselectivity, thereby enabling the implementation of various enzymatic reactions(Datta et al. 2013; Mattiasson 2018). Ventura et al. entrapped chitinase in alginate and zinc oxide nanoparticles (ZnO-NPs) (Ventura et al. 2024); the entrapped chitinase retained its activity for 45 days and exhibited a stronger response in fungal detection compared to free chitinase. Ilyina et al. (Ilyina et al. 2013) microencapsulated chitinase, which maintained its activity for 40 days, whereas the non-encapsulated enzyme retained activity for only 20 days. Furthermore, the microencapsulated chitinase exhibited antifungal activity against Fusarium oxysporum in soil within 40 days and reduced the mortality rate of tomato plants by 70%. Beyond entrapment technology, numerous researchers have immobilized chitinase on different substrates to improve its thermostability and activity. For example, Prasad immobilized a recombinant thermostable fungal chitinase on a phenyl-agarose matrix(Prasad and Palanivelu 2014); the immobilized enzyme displayed significant thermal tolerance at 50°C and higher activity under acidic pH conditions. When the same enzyme was immobilized on glutaraldehyde-crosslinked chitosan beads(Prasad and Palanivelu 2015), it exhibited a broader optimal pH range, greater stability under alkaline pH conditions, and optimal activity at 60°C. Notably, Charoenpol et al. overcame the bottlenecks of free enzyme stability and recyclability through magnetic nanoparticle-based immobilization technology(Charoenpol et al. 2024): the immobilized enzyme retained 39.7% of its activity after 16 cycles of reuse, outperforming similar systems. Additionally, this technology enabled the efficient conversion of chitin waste into high-purity chitooligosaccharides, providing a solution with both scientific innovation and industrial application value in the field of biocatalysis. Entrapment and immobilization technologies have successfully improved the stability and activity of chitinases. To unleash the full potential of chitinases, future research should further optimize entrapment processes, explore more efficient and stable immobilization carriers, and expand the application scope of chitinases.

### Optimization of chitinase synthesis conditions: from fermentation regulation to gene expression

The efficient enzymatic hydrolysis of insoluble chitin relies not only on substrate pretreatment and enzyme molecular engineering but also requires optimization of synthesis conditions to improve the enzyme’s production efficiency and catalytic performance. Fermentation process optimization and gene expression regulation are the core components of this strategy: the former enhances enzyme yield by controlling microbial metabolic networks; the latter achieves efficient heterologous expression of the enzyme by precisely regulating transcriptional levels through promoter engineering. Optimizing fermentation conditions is a conventional approach to enhancing enzyme activity, yet this method not only has limited efficacy in improving activity but also fails to enhance the enzyme’s adaptability to substrates. During fermentation, parameters such as medium formulation (including carbon sources, nitrogen sources, and inorganic salt composition), pH value, fermentation temperature, duration, and oxygen content all significantly affect the yield and activity of the target enzyme. Taking the fermentative production of chitin deacetylase (CDA) as an example, the optimal temperature for CDA production varies among different strains: NaCDA, isolated from *Aquimarinus Nitratireductor* by Chai et al. (2020), exhibits maximum activity at 30°C and retains over 60% of its activity within the temperature range of 25–35°C; However, when the temperature exceeds 45°C or drops below 20°C, its activity drops sharply to below 30%. pH value is also a key factor regulating CDA production(Chai et al. 2020); for instance, the optimal pH for CDA production by *Penicillium herquei* and *Mucor bacilliformis* is 9 and 7.5, respectively. Furthermore, studies on CDA from *Flammulina velutipes*(Pareek et al. 2012) and *Colletotrichum lindemuthianum* have shown that Zn^2^⁺ and Cd^2^⁺ play a crucial role in regulating their catalytic activity. Evidently, the optimization of fermentation conditions is crucial for the efficient production of enzymes and holds significant research value in industrial applications. This study summarizes the optimal fermentation conditions for selected chitinase-producing strains (Table 4).

**Table 4:** Optimum fermentation conditions of chitinase-producing strains

Source	Substrate (colloidal chitin)	Time	Temperature	Activity	References
Chitinilyticum sp.	2%	24 h	50℃	13.32U/mg	Zhao et al. 2023a, b
New chitinase ChiT-7	1%	24 h	45℃	0.63U/mg	Li et al. 2020
A.psychrochitiniphilus492	5.0%	96 h	35℃	335.63U/L	Vasquez et al. 2021
Thermomyces dupontii ITCC9104	0.1%	24 h	70℃	372.57U/L	Kumari et al. 2024
Bacillus thuringiensis Bt028	0.8%	48 h	30℃	10.48U/mL	Xu et al. 2022
Lecanicillium muscariumCCFEE5003	2.0%	5 h	25℃	243U/L	Barghini et al. 2013
A.hydrophila(HS4)	0.3%	24 h	37℃	97.35U/ml	Kuddus et al. 2013
A.punctata(HS6)	0.3%	48 h		82.36U/ml	
Bacillus halodurans C-125(chiA)	NPGlcNAc	18 h	40℃	982.23U/ml	da Silva et al. 2014
Photobacterium sp.(LG-1)	12.0 g/L	120 h	20℃	5.10U/mL	Chen et al. 2021
*Arthrobacter protophormiae*CDA2-2–2	0.5%	84 h	30℃	14.58 U/mL	Zhang et al. 2021a, b
*Bacillus thuringiensis*(Bt-016)	2.5%	48-58 h	30℃	2.7961U/mL	Fu et al. 2019
*Serratia marcescens*(MEW06)	8‰	48 h	31℃	197.32U/mL	Yuan et al. 2018
*Serratia marcescens*(GF-21)	10 g/L	5 h	30℃	4.73U/m L	Zheng et al. 2018
*Humicola grisea*	1%	72 h	60℃	172.47U/L	Kumar et al. 2017
*Cellulosimicrobium cellulans* RS7	1%	48 h	37℃	67.6U/ml	Mahajan et al. 2023
New chitinase P1724	1%	24 h	40℃	2.9U/mg	Dai et al. 2021
*Trichomonas spongiosa* ITCC 8895	1%	48 h	70℃	120U/L	Kumar et al. 2021
*Pseudoalteromonassp*.DL-6 chi C	1%	48 h	30℃	108.56U/mg	Chen et al. 2020
*Bacillus thuringiensis*	5%	6 h	37℃	31 U/mg	Li et al. 2022a, b

### Regulation of chitinase expression: promoter engineering and regulatory element integration

**Fig. 6:**
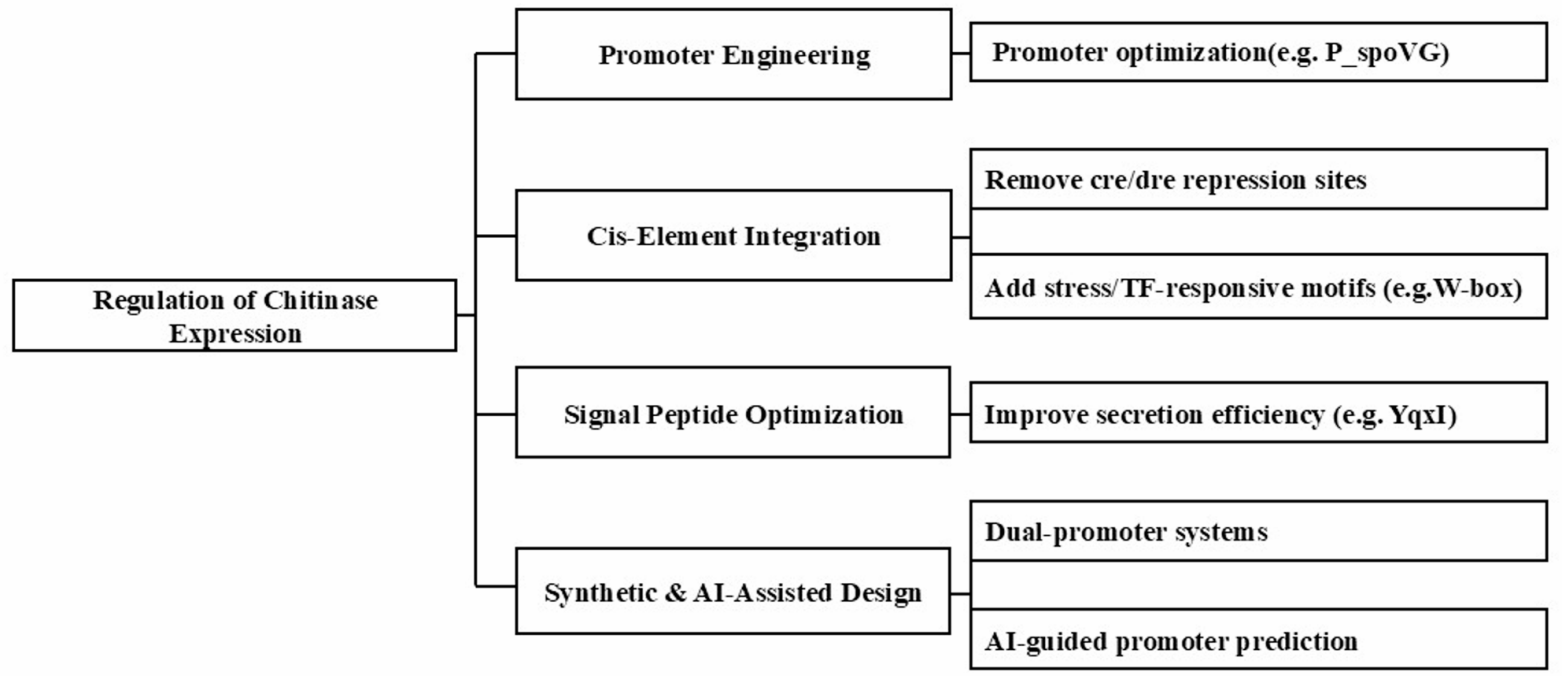
Several expression regulation strategies

The expression of chitinases is governed by a sophisticated network of promoter strength, transcriptional control, and regulatory motifs that collectively determine enzyme yield and secretion efficiency. Although native chitinases often exhibit low activity toward insoluble crystalline chitin, promoter engineering and regulatory element integration can markedly enhance overall catalytic output by increasing active enzyme availability(Fig. 6). Yuan et al. (2023)demonstrated that optimizing both promoter and signal peptide combinations in *Bacillus subtilis* significantly boosted the expression of *BlChiA*, with the SpoVG promoter and YqxI signal peptide increasing activity by 1.33-fold and 1.94-fold, respectively. Similarly, inducible promoters can provide precise temporal regulation—Moradyar et al. (2023)reported 3.2–5.8-fold higher activity in inducible constructs compared with non-induced strains. In plant systems, promoter polymorphisms have also been shown to affect defense-related chitinase expression: in cucumber, a single-nucleotide variant within the CsChi23 promoter alters HD-Zip III transcription factor binding (Bartholomew et al. 2022), while the IbChiA promoter of sweet potato contains pathogen-responsive W-box elements that are up-regulated under fungal stress (Wu et al. 2022). Together, these findings confirm that transcriptional control through promoter architecture and regulatory motifs is a critical determinant of chitinase expression.

Beyond transcriptional optimization, fine-tuning cis-elements and signal peptides further improves expression efficiency. In bacterial hosts, repression through catabolite-responsive sites such as *cre* and *dre* limits chitinase transcription under high-carbon conditions; removal or mutation of these motifs can relieve suppression and enable continuous enzyme production (Martínez et al. 2020; Jiang et al. 2015). Combinatorial strategies—such as dual-promoter systems (e.g. P_spoVG–P_spoVG142) or inducible constructs paired with optimized 5′-UTRs and secretion signals—have yielded up to threefold increases in extracellular enzyme levels (Yao et al. 2023). Collectively, these results demonstrate that regulatory optimization, rather than catalytic modification alone, can substantially enhance chitinase productivity. However, excessive overexpression may saturate secretion pathways, and higher enzyme quantity does not always translate to improved degradation of insoluble substrates. Future advances are expected from AI-assisted promoter prediction and synthetic regulatory-element design, which will enable the rational, data-driven control of chitinase gene expression and its integration with other protein-engineering strategies for industrial biocatalysis.

### Section summary

In summary, traditional enzyme engineering approaches—including rational design, directed evolution, domain fusion, enzyme immobilization, and expression regulation—have collectively advanced chitinase performance in terms of catalytic efficiency, substrate binding, stability, and industrial applicability. Each strategy exhibits distinct strengths: rational design provides mechanistic precision; directed evolution enables empirical exploration; domain fusion significantly enhances activity toward insoluble crystalline chitin; immobilization improves operational stability; and expression regulation ensures efficient production. Nevertheless, most of these approaches remain labor-intensive and rely heavily on trial-and-error processes, particularly when dealing with complex enzyme–substrate interactions and insoluble biopolymers. To provide a clearer comparative overview of these strategies, their mechanisms, advantages, and limitations are summarized in Table 5 below. This comparative evaluation highlights that while conventional engineering methods have achieved substantial progress, they still face limitations in predictive accuracy and efficiency. Consequently, the integration of artificial intelligence (AI) into enzyme design has emerged as a transformative paradigm, enabling data-driven prediction, rational optimization, and de novo enzyme generation. The following section will explore how AI-assisted modeling and generative algorithms are reshaping the directed design of high-activity chitinases.

**Table 5:** Comparative evaluation of major chitinase engineering strategies

**Engineering** **strategy**	**Representative approaches**	**Mechanistic basis**	**Strengths**	**Limitations**
Directed evolution	Error-prone PCR, DNA shuffling, iterative saturation mutagenesis (ISM),	Mimics natural selection through random mutagenesis and high-throughput screening	Does not require structural knowledge; can discover synergistic mutations with unexpected benefits	Labor-intensive and time-consuming; limited enhancement for insoluble crystalline chitin due to screening bias
Rational design	Structure-based site-directed mutagenesis, molecular dynamics simulation, energy minimization, active-site remodeling	Precisely identifies key residues; optimizes catalytic pocket geometry and hydrophobic core; reshapes substrate-binding groove	High precision and reproducibility; mechanism interpretable; can integrate AI tools (e.g. AlphaFold, RosettaDesign) for predictive optimization	Requires high-quality structural information and computational resources; predicted effects may deviate from experiments
Domain fusion	C- or N-terminal fusion of CBM, FnIII, PKD, or CBD domains; multi-domain constructs (e.g. FnIII–ChBD)	Enhances substrate adsorption and local enzyme concentration; stabilizes catalytic domain conformation	Most effective for insoluble and crystalline chitin; adjustable binding strength via domain selection	Large fusion proteins may fold inefficiently; expression yields often reduced; multi-domain interference possible
Enzyme immobilization	Covalent attachment (chitosan beads, SiO₂, Fe₃O₄ nanoparticles), physical adsorption, entrapment (alginate)	Restricts conformational mobility and denaturation; enhances operational stability	Enables enzyme recycling and long-term operation in reactors	Initial catalytic rate may decrease; carrier materials can be costly; immobilization parameters must be optimized
Expression regulation	Strong promoters (T7, P43, AOX1), signal peptide optimization, chaperone co-expression, host system engineering (E. coli, B. subtilis, P. pastoris)	Enhances transcription/translation efficiency and protein folding; reduces inclusion body formation	Boosts enzyme productivity and solubility; facilitates scale-up	Host-dependent optimization required; regulation mechanisms vary across systems

## Development of novel chitinase resources: innovations in high-efficiency mining technologies and AI-assisted design

### High-efficiency enzyme resource development: metagenomics- and database-based strategies

Chitinases are ubiquitously distributed across microorganisms, plants, and animals (Ouyang et al. 2024; Wu et al. 2021). However, traditional isolation and culture-dependent methods remain limited by low efficiency and prolonged screening cycles (Taokaew and Kriangkrai 2023), with approximately 99% of microbial resources in soil still inaccessible by conventional techniques (Pasin et al. 2021).Recent advances in metagenomics and metatranscriptomics have established a comprehensive workflow encompassing environmental sampling → gene library construction → functional screening(Yin et al. 2023; Song et al. 2025). These methods bypass microbial cultivation, enabling direct recovery of chitinase genes from environmental samples such as seawater, marine sediments, corals, and sponges, leading to the discovery of numerous novel endo- and exo-chitinases (Raimundo et al. 2021). This method is particularly advantageous when analyzing metagenomic data from extreme environments (e.g. deep-sea habitats, hot springs, high-salinity and alkaline regions), as it enables effective identification of adaptive enzyme molecules that are recalcitrant to isolation under traditional culture conditions—thereby providing abundant resources for the study of novel enzyme mechanisms and engineering applications(Santos et al. 2025; Neun et al. 2022). The key to efficient enzyme mining lies in the precise capture of functionally active genes while avoiding retrieval of silent or non-functional sequences. For example, Dai et al. (2021) constructed a metagenomic cDNA library from alpine wetland soils of the Qinghai-Tibet Plateau and identified a novel dual-domain chitinase (P1724) with cold adaptability capable of hydrolyzing colloidal and powdered chitin at 4 °C. In parallel, the integration of metagenomics with high-throughput screening has markedly accelerated the discovery of functional biocatalysts (Rembeza et al. 2022).In recent years, AI-assisted annotation and functional prediction have further improved the precision of enzyme mining. Deep-learning models such as AlphaFold3 and ESMFold enable accurate protein structure prediction(Meng et al. 2025), while clustering based on structural similarity facilitates systematic exploration of enzyme families (Huang et al. 2023). These approaches help distinguish catalytically competent chitinases from inactive homologs and pre-screen thermostable or solvent-tolerant candidates (Zhang et al. 2022). For instance, AI-based thermostability prediction tools (e.g. Preoptem) have been used to identify a novel thermophilic chitinase from the marine microbiome with an exceptionally high melting temperature (Tm = 89.65 °C ± 0.22 °C), demonstrating significant industrial potential.

Building upon these AI-driven discovery frameworks, future studies may further focus on constructing structure–function association models for chitinases with high activity toward crystalline or insoluble chitin, enabling systematic exploration of protein databases and metagenomic samples. These advances in AI-guided discovery pave the way for the next stage—rational and generative design of high-activity chitinases.

### Directed design of high-activity chitinases

The integration of artificial intelligence (AI) into enzyme engineering has revolutionized chitinase design, enabling predictive and generative strategies that transcend empirical mutagenesis. Deep-learning models such as AlphaFold2/3 and ESMFold now achieve near-experimental accuracy in chitinase tertiary structure prediction, revealing catalytic-domain organization, substrate-binding clefts, and inter-domain flexibility crucial for crystalline-chitin degradation (Homma et al. 2023; Kyro et al. 2025). Coupling these predictions with molecular dynamics (MD) and hybrid QM/MM simulations allows dynamic visualization of enzyme–substrate interactions and transition-state stabilization, while machine-learning regression models predict properties such as optimal temperature, pH, and substrate affinity from sequence and structural descriptors—accelerating pre-experimental screening(Wu et al. 2024). AI also enhances traditional directed evolution by enabling in silico mutational-landscape prediction. Using supervised and reinforcement learning, mutation combinations likely to enhance activity or stability can be prioritized. Bayesian optimization and graph neural network (GNN)-based residue-interaction models facilitate interpretable exploration of synergistic mutation clusters (Kozome et al. 2024; Oyeleye et al. 2018). For example, researchers conducted computationally guided iterative saturation mutagenesis on chitinase BcChiA1 from Bacillus circulans and introduced transition-state analogs (TSA) to simulate its catalytic mechanism—successfully generating the mutant BcChiA1-ISM. This mutant exhibited a 29.3-fold increase in enzymatic activity relative to the wild type, alongside a marked enhancement in binding capacity toward crystalline chitin (Li et al. 2022a, b).

Beyond optimization, AI now enables de novo enzyme creation. Generative deep models such as ProteinMPNN, ESM-IF1, and diffusion-based architectures can design new chitinase sequences that fold into catalytically competent GH18 or GH19-like scaffolds. Conditioning generative models on target properties (e.g. thermostability, solvent tolerance, or crystalline-chitin binding) allows for the design of tailored enzymes. Subsequent AlphaFold3 validation and energy refinement ensure structural feasibility before experimental synthesis, forming a closed-loop “generate–predict–validate–test” workflow (Yang et al. 2025a, b; Hua et al. 2024) Currently, AI-based Ancestral Sequence Reconstruction (ASR) integrates deep protein-language models (e.g. ESM2, ProtGPT2) with evolutionary inference to predict ancestral chitinases possessing enhanced thermostability and catalytic robustness(Wang et al. 2024a, b, c). Combining ASR with molecular dynamics and stability scoring (e.g. ASSMD framework)(Wang et al. 2024a, b, c) enables rational identification of conserved stabilizing motifs (Zhang et al. 2022; Han et al. 2024a, b). Emerging Explainable AI (XAI) techniques further improve model interpretability by revealing key residues involved in catalysis or substrate binding, while integration with robotic automation and DBTL (Design–Build–Test–Learn) platforms facilitates self-driving enzyme engineering pipelines (Dwivedi et al. 2023; Jeon et al. 2025; Kim et al. 2025).

**Fig. 7:**
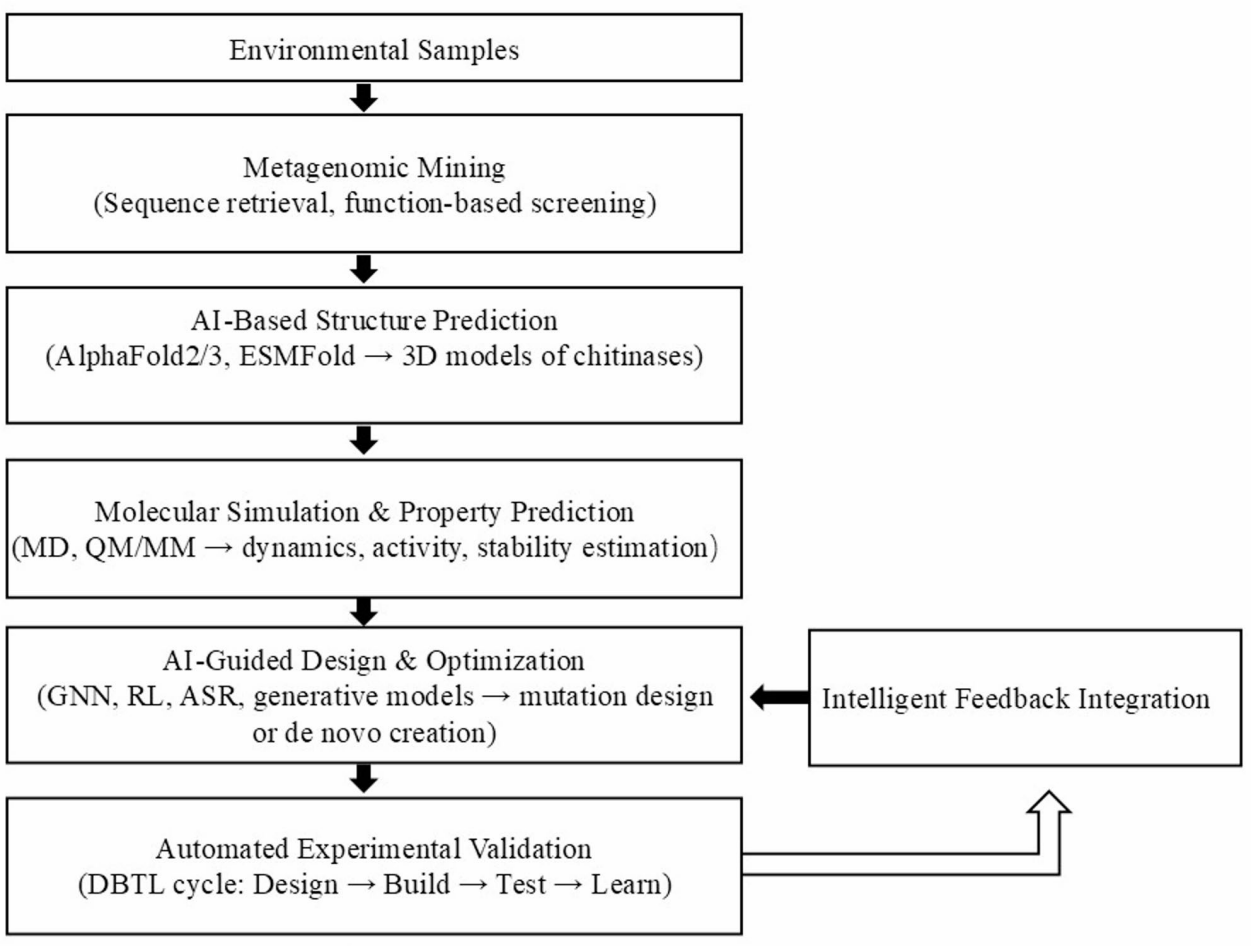
AI-Guided Workflow for Chitinase Discovery and Design(Schematic overview of the AI-assisted workflow for chitinase discovery and engineering. Metagenomic mining retrieves novel chitinase genes from environmental samples, followed by deep-learning–based structure prediction and molecular simulation to assess catalytic and structural features. AI-driven design integrates graph and generative models for mutation optimization or de novo enzyme creation. Experimental validation within an automated DBTL (Design–Build–Test–Learn) cycle feeds results back to the model, forming a self-optimizing loop for intelligent chitinase design.)

Ultimately, the integration of metagenomic mining, structural prediction, and AI-driven design heralds a new paradigm in chitinase research—transforming enzyme engineering from a static, trial-based discipline into a data-centric, self-optimizing intelligence system(Cheng et al. 2025). Unlike traditional workflows that rely on human intuition and stepwise iteration, this emerging paradigm enables a closed-loop evolution of discovery and design, in which algorithms continuously refine hypotheses based on experimental feedback(Fig. 7). By coupling multi-scale simulations, protein language models, and reinforcement-driven optimization under an autonomous DBTL (Design–Build–Test–Learn) framework, AI-guided chitinase engineering will evolve from predicting enzymes to understanding and inventing catalytic principles themselves(Yu et al. 2023). Such a transformation would not only accelerate industrial enzyme discovery but also deepen our mechanistic understanding of polysaccharide degradation—positioning chitinases as a model system for the intelligent design of complex biocatalysts.

## Multi-strategy synergistic system for insoluble chitin degradation

The degradation of insoluble chitin represents a multi-layered catalytic challenge governed by substrate crystallinity, enzyme accessibility, and operational stability(Xue et al. 2021). In recent years, the convergence of molecular engineering, expression optimization, process intensification, and AI-assisted design has promoted a conceptual transition from isolated enhancement toward a multi-strategy synergistic system. Within this framework, individual strategies function as interdependent modules that reinforce one another to overcome the physicochemical limitations of crystalline chitin, establishing a dynamic and sustainable catalytic network.

At the molecular level, site-directed mutagenesis and domain fusion operate in concert to enhance catalytic efficiency and substrate binding. The integration of carbohydrate-binding modules (CBMs) or polysaccharide-interacting linkers improves enzyme contact with crystalline chitin(Sun et al. 2023), thus promoting processive hydrolysis. Structural refinement guided by AI-assisted mining—including the identification of cooperative mutations, flexible loop regions, and linker architectures—further improves structural adaptability and substrate flow(Xie and Warshel 2023; Landwehr et al. 2025). This molecular coupling transforms local activity enhancement into continuous depolymerization of insoluble chitin fibers, forming the foundation for system-level synergy. At the cellular and expression level, promoter regulation and secretion optimization act as amplification interfaces that translate molecular improvements into high-yield enzyme production. Engineered chitinases, often enlarged by domain fusion, impose translational and folding burdens; thus, rational promoter–signal peptide design ensures balanced transcription and proper secretion. AI-directed design accelerates this integration by predicting host–promoter–vector compatibility and optimizing regulatory combinations for efficient synthesis and release(Wang et al. 2023a, b, c; Rafi et al. 2024; Jaganathan et al. 2025). This layer bridges molecular design and process application, maintaining the functional stability–activity balance required for large-scale production. At the process level, enzyme immobilization and reactor configuration convert soluble biocatalysts into robust, recyclable systems suitable for industrial use(Mao et al. 2024). Immobilization preserves enzyme conformation under operational stresses, while engineered thermostable variants exhibit extended half-life and improved carrier interaction(Wei et al. 2023). The synergy between molecular design and immobilization generates a self-reinforcing feedback loop—molecular stability enhances immobilization efficiency, and immobilization in turn protects the engineered conformation. Across these interconnected layers, AI plays a dual role—as an assistant that mines effective sequences, promoters, and carrier matrices, and as a designer that integrates them into predictive and self-optimizing workflows(Landwehr et al. 2025).In this data-informed framework, molecular insights feed into process optimization, and the resulting performance feedback iteratively updates molecular design, completing a closed loop of learning and refinement(Singh et al. 2025; Yang et al. 2025a, b).

**Fig. 8:**
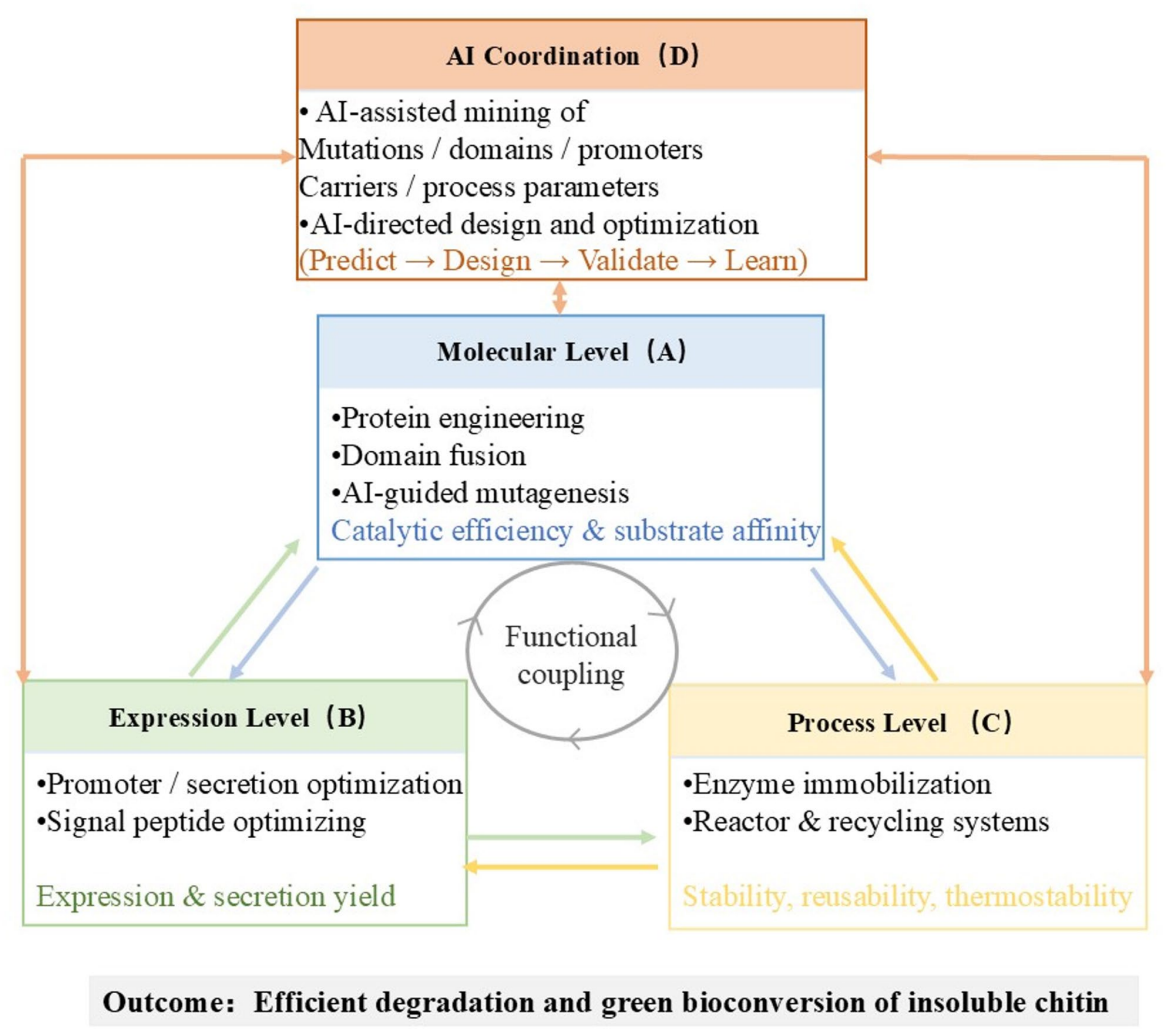
AI-driven multi-strategy synergistic system for efficient degradation and green bioconversion of insoluble chitin. The system integrates molecular engineering. **A** expression optimization. **B** and process stabilization. **C** under the coordination of AI-assisted mining and AI-directed design. **D**. Functional coupling and bidirectional feedback among these layers form a self-reinforcing catalytic loop, in which AI continuously learns from molecular and process data to enhance overall system performance. The outcome is efficient degradation and sustainable bioconversion of crystalline/insoluble chitin, achieving high activity, stability, and recyclability through predictive, data-driven optimization

Collectively, this AI-coordinated multi-strategy system establishes a framework for the efficient degradation and green bioconversion of insoluble chitin. Its performance emerges not from additive improvement but from functional coupling, bidirectional feedback, and multi-scale reinforcement(Fig. 8), providing a sustainable paradigm for chitin bioconversion and industrial enzyme engineering.

## Conclusions

The green bioconversion of insoluble chitin represents a critical strategy for realizing the high-value utilization of crustacean waste. Research in this domain has progressed from structural elucidation, enzymatic mechanism exploration, and chitinase screening/isolation to a phase centered on multi-technological synergy and practical application implementation. This study systematically delineates the degradation-resistant barriers of natural insoluble chitin: its high crystallinity (60–85%) and dense intermolecular hydrogen bond network collectively constitute inherent resistance to enzymatic hydrolysis. It further analyzes the catalytic mechanisms of chitinases from the GH18 and GH19 families, revealing the functional principles of catalytic domains and substrate-binding domains—thereby providing a molecular mechanism-based reference for enzyme engineering modifications. Furthermore, this study summarizes the disruptive effects of physical and chemical pretreatment on structural barriers, as well as the application of strategies (e.g. directed evolution, rational design, substrate-binding domain fusion, and immobilization technology) in enhancing enzymatic hydrolysis efficiency. It also collates the promotional effects of fermentation regulation and gene expression optimization on enzyme synthesis, and examines the application potential of cutting-edge protein modification technologies—including sequence- and structure-based de novo design and ancestral sequence reconstruction (ASR)—in the development of insoluble chitin-degrading enzymes.

Despite significant advancements in chitinase-related research using colloidal chitin as a substrate, the efficient and green conversion of large quantities of natural insoluble chitin still confronts multiple challenges: First, the degradation resistance of natural substrates has not yet been fully overcome, with most existing pretreatment technologies constrained by high energy consumption or excessive costs; second, chitinases exhibit inadequate specific activity and stability toward insoluble chitin, with most high-activity chitinases still relying on colloidal chitin as a substrate—which presents significant cost and environmental pollution challenges in practical industrial applications; furthermore, the integration level of multi-technological synergy remains insufficient, and the full-chain green process (encompassing green pretreatment, efficient enzymatic hydrolysis, and product separation) is not yet mature, leading to poor economic feasibility for large-scale applications. Future research should focus on the in-depth integration of multidisciplinary technologies: On one hand, leveraging AI-assisted design (e.g. integrating AlphaFold3 prediction(Abramson et al. 2024) with molecular dynamics simulations) and metagenomic mining to directionally modify or screen novel chitinases that possess both high thermostability and high activity toward insoluble substrates—thus tackling the core challenge of efficient green biodegradation; On the other hand, advancing innovation in green pretreatment technologies (e.g. developing recyclable ionic liquids(Li et al. 2019) or low-energy physical methods) to reduce pretreatment costs and provide robust support for enzymatic hydrolysis. Additionally, it is essential to strengthen the synergistic application of enzyme engineering strategies: for instance, combining domain fusion with immobilization technology to improve the recycling efficiency of enzymes; optimizing the metabolic networks of strains for target enzyme synthesis via synthetic biology to lower fermentation costs. At the application level, further exploration of the structure–activity relationships of enzymatic hydrolysis products should be conducted to expand their utility in emerging fields (e.g. smart materials and precision medicine). Ultimately, integrating life cycle assessment (LCA) and techno-economic analysis (TEA) into process design will guide the establishment of closed-loop (Morales et al. 2025; Sista et al. 2025), industrial-scale biorefineries that convert chitinous waste into high-value bioproducts. Through enhanced synergy across different levels, the green bioconversion of insoluble chitin can evolve from a laboratory proof-of-concept into a scalable and sustainable manufacturing reality. Such advancements will not only enable the high-value utilization of marine crustacean waste but also foster practical deployment in sectors such as bioplastics, biomedical materials, and agricultural biocontrol—underscoring the industrial significance and translational potential of AI-assisted chitin bioprocessing.

## Data Availability

Not applicable.

## Abbreviations

COSs: Chitooligosaccharides
DES: Deep eutectic solvents
WAXS: Wide-angle X-ray scattering
SANS: Small-angle neutron scattering
UMG: Ultra-micro grinding
HPH: High-pressure homogenization
ICSE: Instantaneous catapult steam explosion
PEF: Pulsed electric field
HCl: Hydrochloric
LPMOs: Lytic polysaccharide monooxygenases
AFM: Atomic force microscopy
SMD: Steered molecular dynamics
3D-AFM: Three-dimensional atomic force microscopy
GH: Glycoside hydrolase
CBMs: CBMs carbohydrate-binding modules
ChBDs: Chitin-binding domains
NMR: Nuclear magnetic resonance
CD: Catalytic domain
CBD: Cellulose-binding domain
ZnO-NPs: Zinc oxide nanoparticles
CDA: Chitin deacetylase
AI: Artificial intelligence
HMM: Hidden Markov model
SODs: Superoxide dismutases
ASR: Ancestral sequence reconstruction
TSA: Transition-state analogs
GGPPS: Geranylgeranyl diphosphate synthase
ASSMD: Ancestral sequence reconstruction with structure and molecular dynamics
LCA: Life cycle assessment
TEA: Techno-economic analysis

## References

[CR1] Abramson J, Adler J, Dunger J, Evans R, Green T, Pritzel A, Jumper JM (2024) Accurate structure prediction of biomolecular interactions with AlphaFold 3. Nature 630(8016):493–500. 10.1038/s41586-024-07487-w 38718835 10.1038/s41586-024-07487-w PMC11168924

[CR2] Akeed Y, Atrash F, Naffaa W (2020) Partial purification and characterization of chitinase produced by *Bacillus licheniformis* B307. Heliyon. 10.1016/j.heliyon.2020.e03858 32395650 10.1016/j.heliyon.2020.e03858 PMC7205749

[CR3] Anil S (2022) Potential medical applications of chitooligosaccharides. Polymers 14(17):3558. 10.3390/polym14173558 36080631 10.3390/polym14173558 PMC9460531

[CR4] Atheena PV, Rajesh KM (2024) Identification and characterization of chitinase producing marine microorganism: unleashing the potential of chitooligosaccharides for bioethanol synthesis. Int J Biol Macromol 265:130846. 10.1016/j.ijbiomac.2024.130846 38492689 10.1016/j.ijbiomac.2024.130846

[CR5] Ashurov, N. S., Abdurazakov, M., Yugay, S. M., Atakhanov, A. A., Gulamjanov, K. A., Turaev, J. I., & Rashidova, S. S. (2022, December). Structure and thermal properties of chitin and chitosan from various sources. In Journal of Physics: Conference Series(Vol. 2388, No. 1, p. 012011). IOP Publishing. 10.1088/1742-6596/2388/1/012011

[CR6] Barghini P, Moscatelli D, Garzillo AMV et al (2013) High production of cold-tolerant chitinases on shrimp wastes in bench-top bioreactor by the Antarctic fungus *Lecanicillium muscarium* CCFEE 5003: bioprocess optimization and characterization of two main enzymes. Enzyme Microb Technol 53(5):331–338. 10.1016/j.enzmictec.2013.07.005 24034432 10.1016/j.enzmictec.2013.07.005

[CR7] Bartholomew ES, Xu S, Zhang Y et al (2022) A chitinase CsChi23 promoter polymorphism underlies cucumber resistance against *Fusarium oxysporum* f. sp. *cucumerinum*. New Phytol 236(4):1471–1486. 10.1111/nph.18463 36068958 10.1111/nph.18463

[CR8] Bhatt P, Joshi S, Bayram GMU, Khati P, Simsek H (2023) Developments and application of chitosan-based adsorbents for wastewater treatments. Environ Res 226:115530. 10.1016/j.envres.2023.115530 36863653 10.1016/j.envres.2023.115530

[CR9] Cao S, Zhu W, Wang W, Wang B, Wei L, Yuan X, Wang H (2025) Mechanochemical conversion of chitin to high-bioactivity oligomers of N-acetyl-D-glucosamine. Carbohydr Polym 354:123289. 10.1016/j.carbpol.2025.123289 39978890 10.1016/j.carbpol.2025.123289

[CR10] Ceballos-Alcantarilla, E., & Merkx, M. (2021). Understanding and applications of Ser/Gly linkers in protein engineering. In Methods in enzymology(Vol. 647, pp. 1–22). Academic Press. 10.1016/bs.mie.2020.12.001 10.1016/bs.mie.2020.12.001 33482985

[CR11] Chokradjaroen C, Niu J, Panomsuwan G, Saito N (2021) Insight on solution plasma in aqueous solution and their application in modification of chitin and chitosan. Int J Mol Sci 22(9):4308. 10.3390/ijms22094308 33919182 10.3390/ijms22094308 PMC8122608

[CR12] Chai J, Hang J, Zhang C, Yang J, Wang S, Liu S, Fang Y (2020) Purification and characterization of chitin deacetylase active on insoluble chitin from *Nitratireductor aquimarinus* MCDA3-3. Int J Biol Macromol 152:922–929. 10.1016/j.ijbio-mac.2020.02.308 32114175 10.1016/j.ijbiomac.2020.02.308

[CR13] Chakravarty J, Edwards TA (2022) Innovation from waste with biomass-derived chitin and chitosan as green and sustainable polymer: a review. Energy Nexus 8:100149. 10.1016/j.nexus.2022.100149

[CR14] Charoenpol A, Crespy D, Schulte A, Suginta W (2024) Immobilized chitinase as effective biocatalytic platform for producing bioactive di-N-acetyl chitobiose from recycled chitin food waste. Bioresour Technol 406:130945. 10.1016/j.biortech.2024.130945 38901749 10.1016/j.biortech.2024.130945

[CR15] Chai, X., Li, J., Liu, J., Chen, X., Li, W., Cheng, M.& Wang, A. (2019). Characteristics and mechanism of a novel chitinase mutant from Trichoderma harzianum with enhanced activity. 10.21203/rs.2.10202/v1

[CR16] Chen J, Yang D, Zhang Y, Yang L, Wang Q, Jiang M, Pan L (2024a) A novel bi-functional cold-adaptive chitinase from *Chitinilyticum aquatile* CSC-1 for efficient synthesis of N-acetyl-D-glucosaminidase. Int J Biol Macromol 259:129063. 10.1016/j.ijbiomac.2023.129063 38159710 10.1016/j.ijbiomac.2023.129063

[CR17] Chen, L. G., Wu, J. W., Zhang, R., et al. (2021). Optimization of Chitinase Production by the Photobacterium sp.LG-1. Science and Technology of Food Industry, 42(1), 6.10.13386/j.issn1002-0306.2019120161

[CR18] Chen, L. G., Zhang, Q. F., Chi, N. Y., et al. (2020). Recombinant Expression of a Cold-Adapted Chitinase Gene in Kluyveromyces lactis and Characterization of Its Enzymatic Properties. Acta Microbiologica Sinica, 60(1), 11.78.10.13343/j.cnki.wsxb.20190129

[CR19] Chen S, Yang Z, Zhong Z, Yu S, Zhou J, Li J, Zhang G (2024b) Ultrahigh-throughput screening-assisted in vivo directed evolution for enzyme engineering. Biotechnol Biofuels Bioprod 17(1):9. 10.1186/s13068-024-02457-w 38254175 10.1186/s13068-024-02457-w PMC10804518

[CR20] Chen X, Pang L, Yang W, Tian H, Yi Y, Xia B (2024c) Enhanced degradation of insoluble chitin: engineering high-efficiency chitinase fusion enzymes for sustainable applications. Bioresour Technol 412:131401. 10.1016/j.biortech.2024.131401 39218366 10.1016/j.biortech.2024.131401

[CR21] Chen X, Zaro JL, Shen WC (2013) Fusion protein linkers: property, design and functionality. Adv Drug Deliv Rev 65(10):1357–1369. 10.1016/j.addr.2012.09.039 23026637 10.1016/j.addr.2012.09.039 PMC3726540

[CR22] Cheng M, Li S, Wang J, Yang X, Duan D, Shao Z (2025) Genome-wide mining of chitinase diversity in the marine diatom *Thalassiosira weissflogii* and functional characterization of a novel GH19 enzyme. Mar Drugs 23(4):144. 10.3390/md23040144 40278265 10.3390/md23040144 PMC12028343

[CR23] da Silva AF, García-Fraga B, López-Seijas J et al (2014) Characterization and optimization of heterologous expression in *Escherichia coli* of the chitinase encoded by the *chiA* gene of *Bacillus halodurans* C-125. Process Biochem 49(10):1622–1629. 10.1016/j.procbio.2014.06.020

[CR24] Dahiya D, Pilli A, Chirra PRR, Sreeramula V, Mogili NV, Ayothiraman S (2022) Morphological and structural characterization of chitin as a substrate for the screening, production, and molecular characterization of chitinase by *Bacillus velezensis*. Environ Sci Pollut Res Int 29(57):86550–86561. 10.1007/s11356-022-22166-x 35895172 10.1007/s11356-022-22166-x

[CR25] Dai Y, Yang F, Liu X et al (2021) The discovery and characterization of a novel chitinase with dual catalytic domains from a Qinghai-Tibetan Plateau wetland soil metagenome. Int J Biol Macromol 188:482–490. 10.1016/j.ijbiomac.2021.07.153 34331981 10.1016/j.ijbiomac.2021.07.153

[CR26] Datta S, Christena LR, Rajaram YRS (2013) Enzyme immobilization: an overview on techniques and support materials. 3 Biotech 3(1):1–9. 10.1007/s13205-012-0071-7 28324347 10.1007/s13205-012-0071-7 PMC3563746

[CR27] Deng JJ, Zhang MS, Li ZW, Lu DL, Mao HH, Zhu MJ, Luo XC (2020) One-step processing of shrimp shell waste with a chitinase fused to a carbohydrate-binding module. Green Chem 22(20):6862–6873. 10.1039/D0GC02611E

[CR28] Deng Y, Liang B, Liang X, Fan S, Zhang Y, Yin Y, Qin Y (2021) Enhancing the solubility of α-chitin in NaOH/urea aqueous solution by synergistic pretreatment of mechanical activation and metal salt. J Mol Liq 339:116756. 10.1016/j.molliq.2021.116756

[CR29] Ding H, Lv L, Wang Z, Liu L (2020) Study on the “glutamic acid-enzymolysis” process for extracting chitin from crab shell waste and its by-product recovery. Appl Biochem Biotechnol 190(3):1074–1091. 10.1007/s12010-019-03139-2 31673937 10.1007/s12010-019-03139-2

[CR30] Dong X, Shi L, Ma S, Chen X, Cao S, Li W, Deng H (2024) Chitin/chitosan nanofibers toward a sustainable future: from hierarchical structural regulation to functionalization applications. Nano Lett 24(39):12014–12026. 10.1021/acs.nanolett.4c02632 39255018 10.1021/acs.nanolett.4c02632

[CR31] Dotto GL, Cunha JM, Calgaro CO, Tanabe EH, Bertuol DA (2015) Surface modification of chitin using ultrasound-assisted and supercritical CO2 technologies for cobalt adsorption. J Hazard Mater 295:29–36. 10.1016/j.jhazmat.2015.04.009 25880046 10.1016/j.jhazmat.2015.04.009

[CR32] Dotto GL, dos Santos JMN, de Moura JM, de Almeida Pinto LA (2016) Ultrasound-assisted treatment of chitin: evaluation of physicochemical characteristics and dye removal potential. E-Polymers 16(1):49–56. 10.1515/epoly-2015-0159

[CR33] Du J, Duan S, Miao J, Zhai M, Cao Y (2021) Purification and characterization of chitinase from *Paenibacillus* sp. Biotechnol Appl Biochem 68(1):30–40. 10.1002/bab.1889 31957084 10.1002/bab.1889

[CR34] Dwivedi R, Dave D, Naik H, Singhal S, Omer R, Patel P, Ranjan R (2023) Explainable AI (XAI): core ideas, techniques, and solutions. ACM Comput Surv 55(9):1–33. 10.1145/3561048

[CR35] Emruzi Z, Aminzadeh S, Karkhane AA, Alikhajeh J, Haghbeen K, Gholami D (2018) Improving the thermostability of *Serratia marcescens* B4A chitinase via G191V site-directed mutagenesis. Int J Biol Macromol 116:64–70. 10.1016/j.ijbiomac.2018.05.014 29733926 10.1016/j.ijbiomac.2018.05.014

[CR36] Eichfeld R, Endeshaw AB, Hellmann MJ, Moerschbacher BM, Zuccaro A (2025) Domain gain or loss in fungal chitinases drives ecological specialization toward antagonism or immune suppression. bioRxiv. 10.1101/2025.06.16.65988639896631

[CR37] Fotodimas I, Vidalis KL, Theodorou JA, Logothetis P, Kanlis G (2025) Sustainable aquaculture through enzymatic hydrolysis of raw chitin from crab by-products: functional fish feeds targeting fish health with implications for human health. Fishes 10(10):514. 10.3390/fishes10100514

[CR38] Fan Y, Fang W, Xiao Y, Yang X, Zhang Y, Bidochka MJ, Pei Y (2007) Directed evolution for increased chitinase activity. Appl Microbiol Biotechnol 76(1):135–139. 10.1007/s00253-007-0996-7 17468866 10.1007/s00253-007-0996-7

[CR39] Farokhi NM, Milani JM, Amiri ZR (2024) Production and comparison of structural, thermal and physical characteristics of chitin nanoparticles obtained by different methods. Sci Rep 14(1):14594. 10.1038/s41598-024-65117-x 38918395 10.1038/s41598-024-65117-x PMC11199498

[CR40] Fu LJ, Yang L, Huang JQ (2019) Optimization of fermentation conditions of chitinase producing Bt-016 strain. J Putian Univ 26(5):6. 10.3969/j.issn.1672-4143.2019.05.013

[CR41] Fernando LD, Dickwella Widanage MC, Penfield J, Lipton AS, Washton N, Latgé JP, Wang T (2021) Structural polymorphism of chitin and chitosan in fungal cell walls from solid-state NMR and principal component analysis. Front Mol Biosci 8:727053. 10.3389/fmolb.2021.727053 34513930 10.3389/fmolb.2021.727053 PMC8423923

[CR42] Guan F, Tian X, Zhang R, Zhang Y, Wu N, Sun J, Huang H (2024) Enhancing the endo-activity of the thermophilic chitinase to yield chitooligosaccharides with high degrees of polymerization. Bioresour Bioprocess 11(1):29. 10.1186/s40643-024-00735-x 38647930 10.1186/s40643-024-00735-x PMC10991111

[CR43] Günal‐Köroğlu D, Karabulut G, Mohammadian F, Can Karaca A, Capanoglu E, Esatbeyoglu T (2025) Production of yeast cell wall polysaccharides‐β‐glucan and chitin by using food waste substrates: biosynthesis, production, extraction, and purification methods. Compr Rev Food Sci Food Saf 24(3):e70161. 10.1111/1541-4337.70161 40183630 10.1111/1541-4337.70161 PMC11970350

[CR44] Gaquere-Parker A, Taylor T, Hutson R, Rizzo A, Folds A, Crittenden S, Arruda A (2018) Low frequency ultrasonic-assisted hydrolysis of starch in the presence of α-amylase. Ultrason Sonochem 41:404–409. 10.1016/j.ultsonch.2017.10.007 29137768 10.1016/j.ultsonch.2017.10.007

[CR45] Gu C, Chen J, Huang X, Jiang Y, Ou N, Yang D, Pan L (2025) The impact of chitinase binding domain truncation on the properties of Ca Chi18B from Chitinilyticum aquatile CSC-1. Mar Drugs 23(3):93. 10.3390/md23030093 40137279 10.3390/md23030093 PMC11943626

[CR46] Guo HZ, Wang D, Yang HT, Wu YL, Li YC, Xia GH, Zhang XY (2024) Heterologous expression and characterization of a pH-stable chitinase from *Micromonospora aurantiaca* with a potential application in chitin degradation. Mar Drugs 22(6):287. 10.3390/md22060287 38921598 10.3390/md22060287 PMC11204758

[CR47] Han S, Xue Y, Yan Q, Jiang Z, Yang S (2024a) Development of a two-enzyme system in Aspergillus niger for efficient production of N-acetyl-β-D-glucosamine from powdery chitin. Bioresour Technol 393:130024. 10.1016/j.biortech.2023.130024 37972902 10.1016/j.biortech.2023.130024

[CR48] Han J, Ullah M, Andoh V, Khan MN, Feng Y, Guo Z, Chen H (2024b) Engineering bacterial chitinases for industrial application: from protein engineering to bacterial strains mutation! A comprehensive review of physical, molecular, and computational approaches. J Agric Food Chem 72(42):23082–23096. 10.1021/acs.jafc.4c06856 39388625 10.1021/acs.jafc.4c06856

[CR49] Hasan I, Gai F, Cirrincione S, Rimoldi S, Saroglia G, Terova G (2023) Chitinase and insect meal in aquaculture nutrition: a comprehensive overview of the latest achievements. Fishes 8(12):607. 10.3390/fishes8120607

[CR50] Halling PJ, Ulijn RV, Flitsch SL (2005) Understanding enzyme action on immobilised substrates. Curr Opin Biotechnol 16(4):385–392. 10.1016/j.copbio.2005.06.006 16005203 10.1016/j.copbio.2005.06.006

[CR51] Hamre AG, Jana S, Reppert NK, Payne CM, Sørlie M (2015) Processivity, substrate positioning, and binding: the role of polar residues in a family 18 glycoside hydrolase. Biochemistry 54(49):7292–7306. 10.1021/acs.biochem.5b00830 26503416 10.1021/acs.biochem.5b00830

[CR52] He, B., Yang, L., Yang, D., Jiang, M., Ling, C., Chen, H., ... & Pan, L. (2022). Biochemical purification and characterization of a truncated acidic, thermostable chitinase from marine fungus for N-acetylglucosamine production. Frontiers in Bioengineering and Biotechnology,10, 1013313. 10.3389/fbioe.2022.1013313 10.3389/fbioe.2022.1013313 PMC9578694 36267443

[CR53] Hibbert EG, Dalby PA (2005) Directed evolution strategies for improved enzymatic performance. Microb Cell Fact 4(1):29. 10.1186/1475-2859-4-29 16212665 10.1186/1475-2859-4-29 PMC1262762

[CR54] Homma F, Huang J, van der Hoorn RA (2023) AlphaFold-multimer predicts cross-kingdom interactions at the plant-pathogen interface. Nat Commun 14(1):6040. 10.1038/s41467-023-41721-9 37758696 10.1038/s41467-023-41721-9 PMC10533508

[CR55] Honda S, Kimura M, Wakita S, Oka Y, Kawakita M, Oyama F, Sakaguchi M (2019) The *Listeria innocua* chitinase LinChi78 has a unique region that is necessary for hydrolytic activity. Appl Microbiol Biotechnol 103(4):1777–1787. 10.1007/s00253-018-9573-5 30610281 10.1007/s00253-018-9573-5

[CR56] Hou F, He L, Ma X, Wang D, Ding T, Ye X, Liu D (2020) Ultrasound enhanced the binding ability of chitinase onto chitin: from an AFM insight. Ultrason Sonochem 67:105117. 10.1016/j.ultsonch.2020.105117 32283493 10.1016/j.ultsonch.2020.105117

[CR57] Hou F, Ma X, Fan L, Wang D, Wang W, Ding T, Liu D (2019) Activation and conformational changes of chitinase induced by ultrasound. Food Chem 285:355–362. 10.1016/j.foodchem.2019.01.180 30797357 10.1016/j.foodchem.2019.01.180

[CR58] Huang H, Zhou G, Cai X, Zhuang M, Xie S (2024) Shell biorefineries: mixed biofuel production from chitin. Green Chem 26(22):11280–11289. 10.1039/D4GC03761H

[CR59] Huang J, Lin Q, Fei H, He Z, Xu H, Li Y, Gao C (2023) Discovery of deaminase functions by structure-based protein clustering. Cell 186(15):3182–3195. 10.1016/j.cell.2023.05.041 37379837 10.1016/j.cell.2023.05.041

[CR60] Hua, C., Lu, J., Liu, Y., Zhang, O., Tang, J., Ying, R., & Zheng, S. (2024). Reaction-conditioned de novo enzyme design with genzyme. arXiv preprint arXiv:2411.16694. 10.48550/arXiv.2411.16694

[CR61] Hudek M, Kubiak-Ossowska K, Johnston K, Ferro VA, Mulheran PA (2023) Chitin and chitosan binding to the α-chitin crystal: a molecular dynamics study. ACS Omega 8(3):3470–3477. 10.1021/acsomega.2c07495 36713729 10.1021/acsomega.2c07495 PMC9878639

[CR62] Irum S, Cilkiz M, Al-Kubaisi N, Elshikh MS, Iqbal R (2025) Genome-wide characterization and expression analysis of the chitinase gene family in chickpea (*Cicer arietinum* L.) for fungal stress resistance. Mol Biol Rep 52(1):871. 10.1007/s11033-025-10937-x 40911260 10.1007/s11033-025-10937-x

[CR63] Ilyina A, León-Joublanc E, Balvantín-García C, Montañez-Sáenz JC, Rodríguez-Garza MM, Segura-Ceniceros EP, Martínez-Hernández JL (2013) Free and encapsulated chitinase and laminarinase as biological agents against Fusarium oxysporum. Afr J Microbiol Res 7(36):4501–4511. 10.5897/AJMR12.2056

[CR64] Jaganathan K, Ersaro N, Novakovsky G, Wang Y, James T, Schwartzentruber J, Farh KKH (2025) Predicting expression-altering promoter mutations with deep learning. Science 389(6760):eads7373. 10.1126/science.ads7373 40440429 10.1126/science.ads7373

[CR65] Jeon S, Sohn YJ, Lee H, Park JY, Kim D, Lee ES, Park SJ (2025) Recent advances in the Design-Build-Test-Learn (DBTL) cycle for systems metabolic engineering of *Corynebacterium glutamicum*. J Microbiol 63(3):e2501021. 10.71150/jm.2501021 40195836 10.71150/jm.2501021 PMC13577085

[CR66] Jiang HK, Lee MN, Tsou JC, Chang KW, Tseng HW, Chen KP, Wang YS (2020) Linker and N-terminal domain engineering of pyrrolysyl-tRNA synthetase for substrate range shifting and activity enhancement. Front Bioeng Biotechnol 8:235. 10.3389/fbioe.2020.00235 32322577 10.3389/fbioe.2020.00235 PMC7156790

[CR67] Jiang K, Li LN, Pan JH, Wang TT, Chen YH, Cai J (2015) YvoA and CcpA repress the expression of chiB in *Bacillus thuringiensis*. Appl Environ Microbiol 81(19):6548–6557. 10.1128/AEM.01549-15 26162881 10.1128/AEM.01549-15 PMC4561717

[CR68] Jiménez-Ortega E, Kidibule PE, Fernández-Lobato M, Sanz-Aparicio J (2021) Structural inspection and protein motions modelling of a fungal glycoside hydrolase family 18 chitinase by crystallography depicts a dynamic enzymatic mechanism. Comput Struct Biotechnol J 19:5466–5478. 10.1016/j.csbj.2021.09.027 34712392 10.1016/j.csbj.2021.09.027 PMC8515301

[CR69] Kadokawa JI (2024) An overview on acylation methods of α-chitin. Int J Biol Macromol 262:130166. 10.1016/j.ijbiomac.2024.130166 38360241 10.1016/j.ijbiomac.2024.130166

[CR70] Kaczmarek MB, Struszczyk-Swita K, Li X, Szczęsna-Antczak M, Daroch M (2019) Enzymatic modifications of chitin, chitosan, and chitooligosaccharides. Front Bioeng Biotechnol 7:243. 10.3389/fbioe.2019.00243 31612131 10.3389/fbioe.2019.00243 PMC6776590

[CR71] Karthick Rajan D, Mohan K, Rajarajeswaran J, Divya D, Kumar R, Kandasamy S, Ramu Ganesan A (2024) β-chitin and chitosan from waste shells of edible mollusks as a functional ingredient. Food Front 5(1):46–72. 10.1002/fft2.326

[CR72] Kawamoto D, Takashima T, Fukamizo T, Numata T, Ohnuma T (2022) A conserved loop structure of GH19 chitinases assists the enzyme function from behind the core-functional region. Glycobiology 32(4):356–364. 10.1093/glycob/cwab117 34939106 10.1093/glycob/cwab117

[CR73] Kawashima S, Ikehata H, Tada C, Ogino T, Kakizaki H, Ikeda M, Matsumiya M (2016) Stomach chitinase from Japanese sardine *Sardinops melanostictus*: purification, characterization, and molecular cloning of chitinase isozymes with a long linker. Mar Drugs 14(1):22. 10.3390/md14010022 26805857 10.3390/md14010022 PMC4728518

[CR74] Khan FI, Bisetty K, Gu KR, Singh S, Permaul K, Hassan MI, Wei DQ (2017) Molecular dynamics simulation of chitinase I from *Thermomyces lanuginosus* SSBP to ensure optimal activity. Mol Simul 43(7):480–490. 10.1080/08927022.2016.1237024

[CR75] Khiyami M, Masmali I (2008) Characteristics of thermostable chitinase enzymes of *Bacillus licheniformis* isolated from Red Palm Weavil Gut. Aust J Basic Appl Sci 2(4):943–948

[CR76] Kitagawa G, Mizuta T, Akamatsu M, Ifuku S (2025) High-yield chitin extraction and nanochitin production from cricket legs. Carbohydrate Polymer Technologies and Applications. 10.1016/j.carpta.2025.100816

[CR77] Kim SK, Park JE, Oh JM, Kim H (2021) Molecular characterization of four alkaline chitinases from three chitinolytic bacteria isolated from a mudflat. Int J Mol Sci 22(23):12822. 10.3390/ijms222312822 34884628 10.3390/ijms222312822 PMC8658002

[CR78] Kim H, Hillson NJ, Cho BK, Sung BH, Lee DH, Kim DM, Lee SG (2025) Abstraction hierarchy to define biofoundry workflows and operations for interoperable synthetic biology research and applications. Nat Commun 16(1):6056. 10.1038/s41467-025-61263-6 40593727 10.1038/s41467-025-61263-6 PMC12214803

[CR79] Kodolova-Chukhontseva VV, Rozova EY, Dresvyanina EN, Nashchekina YA, Dobrovol’skaya IP, Vlasova EN, Morganti P (2023) New composite materials based on chitosan films reinforced with chitin nanofibrils for cosmetic application. Cosmetics 10(2):51. 10.3390/cosmetics10020051

[CR80] Kozome, D., Sljoka, A., & Laurino, P. (2024). Remote loop evolution reveals a complex biological function for chitinase enzymes beyond the active site. Nature Communications,15(1), 3227. 10.1038/s41467-024-47588-8Kuddus, M., & Ahmad, I. Z. (2013). Isolation of novel chitinolytic bacteria and production optimization of extracellular chitinase. Journal of Genetic Engineering and Biotechnology, 11(1), 39–46 10.1038/s41467-024-47588-8 PMC11018821 38622119

[CR81] Kulka K, Sionkowska A (2023) Chitosan based materials in cosmetic applications: a review. Molecules 28(4):1817 36838805 10.3390/molecules28041817 PMC9959028

[CR82] Kumar M, Brar A, Vivekanand V et al (2017) Production of chitinase from thermophilic *Humicola grisea* and its application in production of bioactive chitooligosaccharides. Int J Biol Macromol 104:1641–1647. 10.1016/j.ijbiomac.2017.04.100 28487199 10.1016/j.ijbiomac.2017.04.100

[CR83] Kumar M, Madhuprakash J, Balan V et al (2021) Chemoenzymatic production of chitooligosaccharides employing ionic liquids and *Thermomyces lanuginosus* chitinase. Bioresour Technol 337:125399. 10.1016/j.biortech.2021.125399 34147005 10.1016/j.biortech.2021.125399

[CR84] Kumari R, Kumar M, Dadheech PK et al (2024) Response surface optimization, purification, characterization and short-chain chitooligosaccharides production from an acidic, thermostable chitinase from *Thermomyces dupontii*. Int J Biol Macromol 267:131362. 10.1016/j.ijbiomac.2024.131362 38583843 10.1016/j.ijbiomac.2024.131362

[CR85] Kumari R, Kumar M, Vivekanand V, Pareek N (2023) Chitin biorefinery: a narrative and prophecy of crustacean shell waste sustainable transformation into bioactives and renewable energy. Renew Sustain Energy Rev 184:113595. 10.1016/j.rser.2023.113595

[CR86] Kumari SVG, Pakshirajan K, Pugazhenthi G (2022) Recent advances and future prospects of cellulose, starch, chitosan, polylactic acid and polyhydroxyalkanoates for sustainable food packaging applications. Int J Biol Macromol 221:163–182. 10.1016/j.ijbiomac.2022.08.203 36067847 10.1016/j.ijbiomac.2022.08.203

[CR87] Kyro, G. W., Qiu, T., & Batista, V. S. (2025). A Model-Centric Review of Deep Learning for Protein Design.arXiv preprint arXiv:2502.19173. 10.48550/arXiv.2502.19173Landwehr

[CR88] G. M., Bogart, J. W., Magalhaes, C., Hammarlund, E. G., Karim, A. S., & Jewett, M. C. (2025). Accelerated enzyme engineering by machine-learning guided cell-free expression.Nature communications,16(1), 865 10.1038/s41467-024-55399-0 PMC11747319 39833164

[CR89] Li F, You X, Li Q, Qin D, Wang M, Yuan S, Bi S (2021) Homogeneous deacetylation and degradation of chitin in NaOH/urea dissolution system. Int J Biol Macromol 189:391–397. 10.1016/j.ijbiomac.2021.08.126 34450142 10.1016/j.ijbiomac.2021.08.126

[CR90] Li J, Huang WC, Gao L, Sun J, Liu Z, Mao X (2019) Efficient enzymatic hydrolysis of ionic liquid pretreated chitin and its dissolution mechanism. Carbohydr Polym 211:329–335. 10.1016/j.carbpol.2019.02.027 30824097 10.1016/j.carbpol.2019.02.027

[CR91] Li J, Wang S, Liu C, Li Y, Wei Y, Fu G, Zhang D (2022a) Going beyond the local catalytic activity space of chitinase using a simulation-based iterative saturation mutagenesis strategy. ACS Catal 12(16):10235–10244. 10.1016/j.ijbiomac.2022.08.203

[CR92] Li RK, Hu YJ, Ng TB, Guo BQ, Zhou ZH, Zhao J, Ye XY (2020) Expression and biochemical characterization of a novel chitinase ChiT-7 from the metagenome in the soil of a mangrove tidal flat in China. Int J Biol Macromol 158:1125–1134. 10.1016/j.ijbiomac.2020.04.242 32360969 10.1016/j.ijbiomac.2020.04.242

[CR93] Li R, Hsueh PH, Ulfadillah SA, Wang ST, Tsai ML (2024) Exploring the sustainable utilization of deep eutectic solvents for chitin isolation from diverse sources. Polymers 16(22):3187. 10.3390/polym16223187 39599277 10.3390/polym16223187 PMC11598258

[CR94] Li, X. Y., Deng, X., Jiang, S. J., et al. (2022). C-terminal domain truncation im-proves the activity of chitinase derived from Bacillus thuringiensis. Acta Microbiologica Sinica, 62(4), 62.10.13343/j.cnki.wsxb.20210422

[CR95] Liu F, Chen S, Chen X, Yong B, He B (2024a) Identification of chitinase from *Bacillus velezensis* strain S161 and its antifungal activity against *Penicillium digitatum*. Protein Expr Purif 223:106562. 10.1016/j.pep.2024.106562 39094814 10.1016/j.pep.2024.106562

[CR96] Liu J, Xie J, Wang S, Feng H, Wang G (2025) Thermostability improvement of the chitinase from *Bacillus circulans* for efficient chitin oligosaccharide production via computational design. Biomolecules 15(3):330. 10.3390/biom15030330 40149866 10.3390/biom15030330 PMC11940100

[CR97] Liu Y, Sun G, Liu J, Lou Y, Zhu J, Wang C (2024b) Enzymatic production of diverse N-acetyl chitooligosaccharides employing a novel bifunctional chitinase and its engineered variants. Food Chem 453:139675. 10.1016/j.pep.2024.106562 38781901 10.1016/j.foodchem.2024.139675

[CR98] Liu, Y., Xiao, Y., Ma, A. J., et al. (2022). Effect of combined treatment of ultra-micro grinding and high pressure homogenization on the physicochemical properties and microstructure of chitin.https://www.cabidigitallibrary.org/doi/full/10.5555

[CR99] Ma J, Long Y, Fu J, Shen N, Wang L, Wu S, Deng X (2025) Genome-wide identification and expression analysis of the GH19 chitinase gene family in sea island cotton. Curr Issues Mol Biol 47(8):633. 10.3390/cimb47080633 40864787 10.3390/cimb47080633 PMC12384234

[CR100] Majengbasan OS, Unuofin JO, Daramola MO, Iwarere SA, Semenya K, Odeniyi OA (2025) Chitinous waste depolymerization and biocontrol potential of *Stenotrophomonas maltophilia* 3E chitinase against mycophytopathogens and *Anopheles gambiae* instar 3. Bioresour Technol Rep 30:102095. 10.1016/j.biteb.2025.102095

[CR101] Mahajan G, Sharma V, Gupta R (2024) Chitinase: a potent biocatalyst and its diverse applications. Biocatal Biotransform 42(2):85–109. 10.1080/10242422.2023.2218524

[CR102] Madland E, Crasson O, Vandevenne M, Sørlie M, Aachmann FL (2019) NMR and fluorescence spectroscopies reveal the preorganized binding site in family 14 carbohydrate-binding module from human chitotriosidase. ACS Omega 4(26):21975–21984. 10.1021/acsomega.9b03043 31891077 10.1021/acsomega.9b03043 PMC6933781

[CR103] Madhuprakash J, Dalhus B, Bissaro B, Duhsaki L, Vaaje-Kolstad G, Sørlie M, Eijsink VG (2025) An alkaliphilic chitinase unveils environment-dependent variation in the canonical catalytic machinery of family-18 glycoside hydrolases. Biochemistry 64(10):2291–2305. 10.1021/acs.biochem.5c00082 40314600 10.1021/acs.biochem.5c00082

[CR104] Mahajan G, Chauhan V, Sharma V et al (2023) Combining in vitro and in silico studies to unravel the antifungal potential of chitinase from a marine bacterial isolate *Cellulosimicrobium cellulans* RS7. Process Biochem 134:88–100. 10.1016/j.procbio.2023.09.024

[CR105] Mao S, Jiang J, Xiong K, Chen Y, Yao Y, Liu L, Li X (2024) Enzyme engineering: performance optimization, novel sources, and applications in the food industry. Foods 13(23):3846. 10.3390/foods13233846 39682920 10.3390/foods13233846 PMC11639928

[CR106] Martinez EA, Boer H, Koivula A, Samain E, Driguez H, Armand S, Cottaz S (2012) Engineering chitinases for the synthesis of chitin oligosaccharides: catalytic amino acid mutations convert the GH-18 family glycoside hydrolases into transglycosylases. J Mol Catal B Enzym 74(1–2):89–96. 10.1016/j.molcatb.2011.09.003

[CR107] Martínez-Ranz M, Kidibule PE, Jiménez-Ortega E, Valcárcel J, Vázquez JA, Sanz-Aparicio J, Fernández-Lobato M (2025) Boosting biocatalytic efficiency: engineering of chitinase Chit33 with chitin and cellulose binding domains for sustainable chitin conversion. J Agric Food Chem 73(18):11121–11131. 10.1021/acs.jafc.4c10364 40279401 10.1021/acs.jafc.4c10364 PMC12129251

[CR108] Martínez-Zavala SA, Barboza-Pérez UE, Hernández-Guzmán G, Bideshi DK, Barboza-Corona JE (2020) Chitinases of *Bacillus thuringiensis*: phylogeny, modular structure, and applied potentials. Front Microbiol 10:3032. 10.3389/fmicb.2019.03032 31993038 10.3389/fmicb.2019.03032 PMC6971178

[CR109] Matroodi S, Motallebi M, Mousavi A (2024) Enhanced antifungal activity of sugarcane cv. NCo310 expressing chimeric chitinase 42. Physiol Mol Plant Pathol 133:102341. 10.1016/j.pmpp.2024.102341

[CR110] Mattiasson, B. (2018). Immobilization methods. In Immobilized cells and organelles (pp. 3–26). CRC Press. 10.1201/9781351073394

[CR111] Meng G, Li L, Wang L, Zhang Y, Zhang L, Ji J, Jiang L (2025) Computational mining and redesign of superoxide dismutase with activity-thermostability improvement. Int J Biol Macromol 307:141871. 10.1016/j.ijbiomac.2025.141871 40058429 10.1016/j.ijbiomac.2025.141871

[CR112] Menghiu G, Ostafe V, Prodanović R, Fischer R, Ostafe R (2021) A high-throughput screening system based on fluorescence-activated cell sorting for the directed evolution of chitinase A. Int J Mol Sci 22(6):3041. 10.3390/ijms22063041 33809788 10.3390/ijms22063041 PMC8002391

[CR113] Minguet-Lobato M, Cervantes FV, Míguez N, Plou FJ, Fernández-Lobato M (2024) Chitinous material bioconversion by three new chitinases from the yeast *Mestchnikowia pulcherrima*. Microb Cell Fact 23(1):31. 10.1186/s12934-024-02300-9 38245740 10.1186/s12934-024-02300-9 PMC10799394

[CR114] Minguet-Lobato M, Fernández-García Á, Jiménez-Ortega E, Cervantes FV, Sánchez-Pozo P, Plou FJ, Fernández-Lobato M (2025) Structural-mechanistic insights and performance engineering of chitinase MpChit35 for tailored chito-oligosaccharide production. Int J Biol Macromol. 10.1016/j.ijbiomac.2025.146538 40763848 10.1016/j.ijbiomac.2025.146538

[CR115] Moradyar, M., Zamani, M., Motallebi, M., et al. (2023). Analysis of the effect of chimeric chitinase expressed by synthetic promoter in T2 generation of transgenic canola. Journal of Genetic Resources, 9(1), 48–58.10.22080/jgr.2023.24506.1336

[CR116] Morales-Gutierrez G, Marulanda-Cardona V (2025) Process model and comparative life cycle assessment (LCA) of a biorefinery concept based on fractionated subcritical water hydrolysis for sugar cane trash valorization. Biomass Bioenerg 196:107740. 10.1016/j.biombioe.2025.107740

[CR117] Nagata T, Shinya S, Ohnuma T, Fukamizo T (2021) Multi-functionality of a tryptophan residue conserved in substrate-binding groove of GH19 chitinases. Sci Rep 11(1):2494. 10.1038/s41598-021-81903-3 33510258 10.1038/s41598-021-81903-3 PMC7844276

[CR118] Nakamura A, Okazaki KI, Furuta T, Sakurai M, Iino R (2018) Processive chitinase is Brownian monorail operated by fast catalysis after peeling rail from crystalline chitin. Nat Commun 9(1):3814. 10.1038/s41467-018-06362-3 30232340 10.1038/s41467-018-06362-3 PMC6145945

[CR119] Navarro P J S. (2022).Insects as alternative sources of value-added by-products: A study of different extraction approaches and characterization of chitin and chitosan from two edible insect species (T. molitor and A. domesticus) and by-products (Master's thesis, Universidade da Beira Interior (Portugal)).

[CR120] Neeraja C, Moerschbacher B, Podile AR (2010) Fusion of cellulose binding domain to the catalytic domain improves the activity and conformational stability of chitinase in *Bacillus licheniformis* DSM13. Bioresour Technol 101(10):3635–3641. 10.1016/j.biortech.2009.12.118 20097556 10.1016/j.biortech.2009.12.118

[CR121] Nestl BM, Hauer B (2014) Engineering of flexible loops in enzymes. ACS Catal 4(9):3201–3211. 10.1021/cs500325p

[CR122] Neun S, Brear P, Campbell E, Tryfona T, El Omari K, Wagner A, Hollfelder F (2022) Functional metagenomic screening identifies an unexpected β-glucuronidase. Nat Chem Biol 18(10):1096–1103. 10.1038/s41589-022-01071-x 35799064 10.1038/s41589-022-01071-x

[CR123] Ngasotter S, Sampath L, Xavier KM (2022) Nanochitin: an update review on advances in preparation methods and food applications. Carbohydr Polym 291:119627. 10.1016/j.carbpol.2022.119627 35698419 10.1016/j.carbpol.2022.119627

[CR124] Ngasotter S, Xavier KM, Meitei MM, Waikhom D, Pathak J, Singh SK (2023) Crustacean shell waste derived chitin and chitin nanomaterials for application in agriculture, food, and health–a review. Carbohydrate Polymer Technologies and Applications 6:100349. 10.1016/j.carpta.2023.100349

[CR125] Ni H, Zeng S, Qin X, Sun X, Zhang S, Zhao X, Li L (2015) Molecular docking and site-directed mutagenesis of a Bacillus thuringiensis chitinase to improve chitinolytic, synergistic lepidopteran-larvicidal and nematicidal activities. Int J Biol Sci 11(3):304. 10.7150/ijbs.10632 25678849 10.7150/ijbs.10632 PMC4323370

[CR126] Niedzialek D, Wieczorek G, Drzewicka K, Antosiewicz A, Milewski M, Bartoszewicz A, Zasłona Z (2025) Elucidation of the catalytic apparatus and mechanism of human Chitotriosidase-1. ACS Catal 15:16748–16761. 10.1021/acscatal.5c00507 41063804 10.1021/acscatal.5c00507 PMC12502790

[CR127] Ohnuma T, Umemoto N, Kondo K, Numata T, Fukamizo T (2013) Complete subsite mapping of a “loopful” GH19 chitinase from rye seeds based on its crystal structure. FEBS Lett 587(16):2691–2697. 10.1016/j.febslet.2013.07.008 23871710 10.1016/j.febslet.2013.07.008

[CR128] Ohnuma T, Umemoto N, Nagata T, Shinya S, Numata T, Taira T, Fukamizo T (2014) Crystal structure of a “loopless” GH19 chitinase in complex with chitin tetrasaccharide spanning the catalytic center. Biochimica Et Biophysica Acta (BBA)-Proteins and Proteomics 1844(4):793–802. 10.1016/j.bbapap.2014.02.013 24582745 10.1016/j.bbapap.2014.02.013

[CR129] Orlando M, Buchholz PC, Lotti M, Pleiss J (2021) The GH19 engineering database: sequence diversity, substrate scope, and evolution in glycoside hydrolase family 19. PLoS ONE 16(10):e0256817. 10.1371/journal.pone.0256817 34699529 10.1371/journal.pone.0256817 PMC8547705

[CR130] Ouyang B, Wang G, Hu Z, Liu Q, Zhao W, Zhao X (2025) A novel directed evolution approach for co-evolution of β-glucosidase activity and organic acid tolerance. J Biotechnol 401:1–10. 10.1016/j.jbiotec.2025.02.009 39983995 10.1016/j.jbiotec.2025.02.009

[CR131] Ouyang, Y. Y., Lin, M., Wang, Q. Z., Lu, B., Lü, X. Y., &Huang, J., et al. (2024). Gene cloning, expression, and enzymatic properties analysis of chitinase from Microbulbifer thermotolerans YLW106. Guangxi Sciences, 31(4), 731–741.10.13656/j.cnki.gxkx.20240416.001

[CR132] Oyeleye A, Normi YM (2018) Chitinase: diversity, limitations, and trends in engineering for suitable applications. Biosci Rep 38(4):BSR2018032300. 10.1042/BSR20180323 30042170 10.1042/BSR20180323 PMC6131217

[CR133] Pan, M., Xu, X., Liu, Y., Li, J., Lü, X., Du, G., & Liu, L. (2019). Directed evolution of chitinase Chisb and biosynthesis of chitooligosaccharides.Sheng wu Gong Cheng xue bao= Chinese Journal of Biotechnology,35(9), 1787–1796. 10.13345/j.cjb.190069Pan 10.13345/j.cjb.190069 31559759

[CR134] M., Li, J., Lv, X., Du, G., & Liu, L. (2019). Molecular engineering of chitinase from Bacillus sp. DAU101 for enzymatic production of chitooligosaccharides. Enzyme and microbial technology,124, 54–62 10.1016/j.enzmictec.2019.01.012 30797479

[CR135] Pareek N, Vivekanand V, Saroj S, Sharma AK, Singh RP (2012) Purification and characterization of chitin deacetylase from *Penicillium oxalicum* SAEM-51. Carbohydr Polym 87(2):1091–1097. 10.1016/j.carbpol.2011.08.041 10.1016/j.carbpol.2013.04.005 23768582

[CR136] Pasin TM, de Oliveira TB, Scarcella ASDA, Polizeli MDLTDM, Guazzaroni ME (2021) Perspectives on expanding the repertoire of novel microbial chitinases for biological control. J Agric Food Chem 69(11):3284–3288. 10.1021/acs.jafc.1c00219 33720714 10.1021/acs.jafc.1c00219

[CR137] Piekarska K, Sikora M, Owczarek M, Jóźwik-Pruska J, Wiśniewska-Wrona M (2023) Chitin and chitosan as polymers of the future—obtaining, modification, life cycle assessment and main directions of application. Polymers (Basel) 15(4):793. 10.3390/polym15040793 36850077 10.3390/polym15040793 PMC9959150

[CR138] Pongsupasa, V., Anuwan, P., Maenpuen, S., & Wongnate, T. (2021). Rational-design engineering to improve enzyme thermostability. In Enzyme engineering: methods and protocols (pp. 159–178). New York, NY: Springer US. 10.1007/978-1-0716-1826-4_9 10.1007/978-1-0716-1826-4_9 34813064

[CR139] Pohling J, Hawboldt K, Dave D (2022) Comprehensive review on pre-treatment of native, crystalline chitin using non-toxic and mechanical processes in preparation for biomaterial applications. Green Chem 24(18):6790–6809. 10.1039/D2GC01968J

[CR140] Poria V, Rana A, Kumari A, Grewal J, Pranaw K, Singh S (2021) Current perspectives on chitinolytic enzymes and their agro-industrial applications. Biology 10:1319. 10.3390/biology10121319 34943233 10.3390/biology10121319 PMC8698876

[CR141] Prakash NAU, Jayanthi M, Sabarinathan R, Kangueane P, Mathew L, Sekar K (2010) Evolution, homology conservation, and identification of unique sequence signatures in gh19 family chitinases. J Mol Evol 70(5):466–478. 10.1007/s00239-010-9345-z 20480157 10.1007/s00239-010-9345-z

[CR142] Prasad M, Palanivelu P (2014) A novel method for the immobilization of a thermostable fungal chitinase and the properties of the immobilized enzyme. Biotechnol Appl Biochem 61(4):441–445. 10.1002/bab.1179 24237246 10.1002/bab.1179

[CR143] Prasad M, Palanivelu P (2015) Immobilization of a thermostable, fungal recombinant chitinase on biocompatible chitosan beads and the properties of the immobilized enzyme. Biotechnol Appl Biochem 62(4):523–529. 10.1002/bab.1283 25195976 10.1002/bab.1283

[CR144] Psarianos M, Dimopoulos G, Ojha S, Cavini ACM, Bußler S, Taoukis P, Schlüter OK (2022) Effect of pulsed electric fields on cricket (*Acheta domesticus*) flour: extraction yield (protein, fat and chitin) and techno-functional properties. Innov Food Sci Emerg Technol 76:102908. 10.1016/j.ifset.2021.102908

[CR145] Qu M, Watanabe-Nakayama T, Sun S, Umeda K, Guo X, Liu Y, Yang Q (2020) High-speed atomic force microscopy reveals factors affecting the processivity of chitinases during interfacial enzymatic hydrolysis of crystalline chitin. ACS Catal 10(22):13606–13615. 10.1021/acscatal.0c02751

[CR146] Rabadiya D, Behr M (2024) The biology of insect chitinases and their roles at chitinous cuticles. Insect Biochem Mol Biol 165:10407. 10.1016/j.ibmb.2024.104071 10.1016/j.ibmb.2024.104071 38184175

[CR147] Rasweefali MK, Nayana A, Rahman MR, Habeebrehman H, Sabu S (2025) Influence of chemical concentrations on the physicochemical, structural, functional and color characteristics of chitin isolated from Arabian red shrimp (Aristeus alcocki). Sustainable Chemistry for the Environment 10:100233. 10.1016/j.scenv.2025.100233

[CR148] Rafi AM, Nogina D, Penzar D, Lee D, Lee D, Kim N, de Boer CG (2024) A community effort to optimize sequence-based deep learning models of gene regulation. Nat Biotechnol. 10.1038/s41587-024-02414-w 39394483 10.1038/s41587-024-02414-w PMC12339383

[CR149] Raimundo I, Silva R, Meunier L, Valente SM, Lago-Lestón A, Keller-Costa T, Costa R (2021) Functional metagenomics reveals differential chitin degradation and utilization features across free-living and host-associated marine microbiomes. Microbiome 9:1–18. 10.1186/s40168-020-00970-2 33583433 10.1186/s40168-020-00970-2 PMC7883442

[CR150] Rana AK, Gupta VK, Hart P, Scarpa F, Thakur VK (2024) Sustainable MXene-chitosan/chitin composites for interdisciplinary applications in water purification, bio-medical, bio-sensing and electronic fields. Materials Today Sustainability 25:100671. 10.1016/j.mtsust.2024.100671

[CR151] Reddy Chichili VP, Kumar V, Sivaraman J (2013) Linkers in the structural biology of protein–protein interactions. Protein Sci 22(2):153–167. 10.1002/pro.2206 23225024 10.1002/pro.2206 PMC3588912

[CR152] Rembeza E, Boverio A, Fraaije MW, Engqvist MK (2022) Discovery of two novel oxidases using a high‐throughput activity screen. ChemBioChem 23(2):e202100510. 10.1002/cbic.202100510 34709726 10.1002/cbic.202100510 PMC9299179

[CR153] Salavati M (2023) Mechanical properties of α-chitin and chitosan biocomposite: a molecular dynamic study. J Compos Sci 7(11):464. 10.3390/jcs7110464

[CR154] Sagar BM, Islam MM, Habib ML, Ahmed S, Hossain MS (2025) Utilization of natural and waste sources for synthesis of cellulose, chitin, and chitosan for a suitable environment. RSC Adv 15(32):26276–26301. 10.1039/D5RA02896E 40703073 10.1039/d5ra02896e PMC12284771

[CR155] Sampath L, Ngasotter S, Layana P, Balange AK, Nayak BB, Xavier KM (2022) Effect of chemical treatment duration on physicochemical, rheological, and functional properties of colloidal chitin. Food Hydrocolloids for Health 2:100091. 10.1016/j.fhfh.2022.100091

[CR156] Santos CA, Morais MA, Mandelli F, Lima EA, Miyamoto RY, Higasi PM, Murakami MT (2025) A metagenomic ‘dark matter’enzyme catalyses oxidative cellulose conversion. Nature. 10.1038/s41586-024-08553-z 39939775 10.1038/s41586-024-08553-z PMC11946906

[CR157] Selvaraj T, Babu MM, Vincent SP, Citarasu T, Punitha MSJ, Das SA, Lipton AP (2009) Mutagenetic study on chitinase producing Chi-a gene from chromobacterium Sp. strain C-61 expressed in *Escherichia coli* XL-1 blue. Int J Biotechnol Biochem 5(1):63–84

[CR158] Selvoski C, Lobarbio CF, Plowman-Holmes M, Bell P, Chambers B, Cumming M (2025) Extraction, quantification, and characterization of chitin from marine biofouling organisms amphipods (Jassa sp.) and hydroids (Coryne sp.). Polysaccharides 6(4):87. 10.3390/polysaccharides6040087

[CR159] Sherali K (2025) POLYMORPHIC CHARACTERIZATION OF CHITIN EXTRACTED FROM SELECTED INSECT SPECIES. Universum: Xимия и Биoлoгия 3(7 (133)):24–28. 10.32743/UniChem.2025.133.7.20478

[CR160] Shen CR, Chen YS, Yang CJ, Chen JK, Liu CL (2010) Colloid chitin azure is a dispersible, low-cost substrate for chitinase measurements in a sensitive, fast, reproducible assay. SLAS Discov 15(2):213–217. 10.1177/1087057109355057 10.1177/1087057109355057 20042532

[CR161] Shimahara, K., & Takiguchi, Y. (1988). Preparation of crustacean chitin. In Methods in enzymology(Vol. 161, pp. 417–423). Academic Press. 10.1016/0076-6879(88)61049-4

[CR162] Sista Kameshwar AK (2025) CAZyXplorer: a shiny application for cost-effective preliminary screening of microbial strains to advance enzyme discovery in biorefining and biotechnology. bioRxiv. 10.1101/2025.10.13.679092

[CR163] Sidar A, Albuquerque ED, Voshol GP, Ram AF, Vijgenboom E, Punt PJ (2020) Carbohydrate binding modules: diversity of domain architecture in amylases and cellulases from filamentous microorganisms. Front Bioeng Biotechnol 8:871. 10.3389/fbioe.2020.00871 32850729 10.3389/fbioe.2020.00871 PMC7410926

[CR164] Sivaramakrishna D, Bhuvanachandra B, Nadendla SR, Podile AR (2020) Efficient conversion of α-chitin by multi-modular chitinase from *Chitiniphilus shinanonensis* with KOH and KOH-urea pretreatment. Carbohydr Polym 250:116923. 10.1016/j.carbpol.2020.116923 33049837 10.1016/j.carbpol.2020.116923

[CR165] Singh N, Lane S, Yu T, Lu J, Ramos A, Cui H, Zhao H (2025) A generalized platform for artificial intelligence-powered autonomous enzyme engineering. Nat Commun 16(1):5648. 10.1038/s41467-025-61209-y 40595587 10.1038/s41467-025-61209-y PMC12215622

[CR166] Song Z, Zhang Q, Wu W, Pu Z, Yu H (2023) Rational design of enzyme activity and enantioselectivity. Front Bioeng Biotechnol 11:1129149. 10.3389/fbioe.2023.1129149 36761300 10.3389/fbioe.2023.1129149 PMC9902596

[CR167] Song, R. H., Zhu, D., Yang, Z. Q., Li, J. L., Yang, Z. F., Lv, Z. H., Yin, Y. R. (2025). Characterization of a GH10 family thermophilic, alkali-and salt-tolerant xylanase from Xinjiang salt lake.Enzyme and Microbial Technology, 110693. 10.1016/j.enzmictec.2025.110693 10.1016/j.enzmictec.2025.110693 40505473

[CR168] Songsiriritthigul C, Pesatcha P, Eijsink VG, Yamabhai M (2009) Directed evolution of a *Bacillus* chitinase. Biotechnol J 4(4):501–509. 10.1002/biot.200800258 19370717 10.1002/biot.200800258

[CR169] Spiwok V (2017) CH/π interactions in carbohydrate recognition. Molecules 22(7):1038. 10.3390/molecules22071038 28644385 10.3390/molecules22071038 PMC6152320

[CR170] Stavros P, Malecki PH, Theodoridou M, Rypniewski W, Vorgias CE, Nounesis G (2015) The stability of the TIM-barrel domain of a psychrophilic chitinase. Biochem Biophys Rep 3:108–116. 10.1016/j.bbrep.2015.07.016 29124173 10.1016/j.bbrep.2015.07.016 PMC5668695

[CR171] Su H, Gao L, Sun J, Mao X (2021) Engineering a carbohydrate binding module to enhance chitinase catalytic efficiency on insoluble chitinous substrate. Food Chem 355:129462. 10.1016/j.foodchem.2021.129462 33848938 10.1016/j.foodchem.2021.129462

[CR172] Suginta W, Sirimontree P, Sritho N, Ohnuma T, Fukamizo T (2016) The chitin-binding domain of a GH-18 chitinase from *Vibrio harveyi* is crucial for chitin-chitinase interactions. Int J Biol Macromol 93:1111–1117. 10.1016/j.ijbiomac.2016.09.066 27667544 10.1016/j.ijbiomac.2016.09.066

[CR173] Sulthan R, Reghunadhan A, Aravind R, Rajeev S, Shankar B, Sambhudevan S (2025) Unveiling the multifunctionality of deep eutectic solvents: their role as solvents, extracting, and curing agents in β-chitin nanowhisker/DGEBA epoxy composites—a comparative study with α-chitin. Int J Biol Macromol. 10.1016/j.ijbiomac.2025.143345 40268036 10.1016/j.ijbiomac.2025.143345

[CR174] Sun B, Zhao X, Xu B, Su E, Kovalevsky A, Shen Q, Wan Q (2023) Discovering and designing a chimeric hyperthermophilic chitinase for crystalline chitin degradation. ACS Sustain Chem Eng 11(12):4690–4698. 10.1021/acssuschemeng.2c07050

[CR175] Sun X, Li Y, Tian Z, Qian Y, Zhang H, Wang L (2019) A novel thermostable chitinolytic machinery of *Streptomyces* sp. F-3 consisting of chitinases with different action modes. Biotechnol Biofuels 12(1):136. 10.1186/s13068-019-1472-1 31171937 10.1186/s13068-019-1472-1 PMC6545677

[CR176] Tanaka J, Fukamizo T, Ohnuma T (2017) Enzymatic properties of a GH19 chitinase isolated from rice lacking a major loop structure involved in chitin binding. Glycobiology 27(5):477–485. 10.1093/glycob/cwx016 28204489 10.1093/glycob/cwx016

[CR177] Taokaew S, Kriangkrai W (2023) Chitinase-assisted bioconversion of chitinous waste for development of value-added chito-oligosaccharides products. Biology 12(1):87. 10.3390/biology12010087 36671779 10.3390/biology12010087 PMC9855443

[CR178] Tian, X. Q., Lu, H. Q., Wu, N. F., Tian, J., & Guan, F. F. (2023). Catalytic properties of the active pocket key tryptophan on chitinase chi304. https://www.nkdb.net/EN/10.13304/j.nykjdb.2021.0877

[CR179] Tian Z, Wang S, Hu X, Zhang Z, Liang L (2018) Crystalline reduction, surface area enlargement and pore generation of chitin by instant catapult steam explosion. Carbohydr Polym 200:255–261. 10.1016/j.carbpol.2018.07.075 30177165 10.1016/j.carbpol.2018.07.075

[CR180] Tjoelker LW, Gosting L, Frey S, Hunter CL, Le Trong H, Steiner B, Gray PW (2000) Structural and functional definition of the human chitinase chitin-binding domain. J Biol Chem 275(1):514–520. 10.1074/jbc.275.1.514 10617646 10.1074/jbc.275.1.514

[CR181] Tokunaga Y, Nagata T, Kondo K, Katahira M, Watanabe T (2020) NMR elucidation of nonproductive binding sites of lignin models with carbohydrate-binding module of cellobiohydrolase I. Biotechnol Biofuels 13(1):164. 10.1186/s13068-020-01805-w 33042221 10.1186/s13068-020-01805-w PMC7541279

[CR182] Topić Popović N, Lorencin V, Strunjak-Perović I, Čož-Rakovac R (2023) Shell waste management and utilization: mitigating organic pollution and enhancing sustainability. Appl Sci 13(1):623. 10.3390/app13010623

[CR183] Tsivileva, O., Pozdnyakov, A., & Ivanova, A. (2021). Polymer nanocomposites of selenium biofabricated using fungi. Molecules, 26(12), 3657.10.3390/molecules26123657 10.3390/molecules26123657 PMC8232642 34203966

[CR184] Uchiyama, T., Katouno, F., Nikaidou, N., Nonaka, T., Sugiyama, J., & Watanabe, T. (2001). Roles of the exposed aromatic residues in crystalline chitin hydrolysis by chitinase A from Serratia marcescens2170. Journal of Biological Chemistry, 276(44), 41343–41349.10.1074/jbc.M103610200 10.1074/jbc.M103610200 11522778

[CR185] Umemoto N, Ohnuma T, Osawa T, Numata T, Fukamizo T (2015) Modulation of the transglycosylation activity of plant family GH18 chitinase by removing or introducing a tryptophan side chain. FEBS Lett 589(18):2327–2333. 10.1074/jbc.M103610200 26216755 10.1016/j.febslet.2015.07.018

[CR186] Uni, F., Lee, S., Yatsunami, R., Fukui, T., & Nakamura, S. (2012). Mutational analysis of a CBM family 5 chitin-binding domain of an alkaline chitinase from Bacillus sp. J813. Bioscience, biotechnology, and biochemistry, 76(3), 530–535. 10.1271/bbb.110835 10.1271/bbb.110835 22451396

[CR187] Van Aalten, D. M. F., Komander, D., Synstad, B., Gåseidnes, S., Peter, M. G., & Eijsink, V. G. H. (2001). Structural insights into the catalytic mechanism of a family 18 exo-chitinase.Proceedings of the National Academy of Sciences, 98(16), 8979–8984. 10.1073/pnas.151103798 10.1073/pnas.151103798 PMC55359 11481469

[CR188] Vasquez, Y. M. S. C., Gomes, M. B., e Silva, T. R., et al. (2021). Cold-adapted chitinases from Antarctic bacteria: taxonomic assessment and enzyme production optimization. Biocatalysis and Agricultural Biotechnology, 34, 102029.10.1016/j.bcab.2021.102029

[CR189] Vasquez, Y. M. S. C., Cueva-Yesquen, L. G., Duarte, A. W. F., Rosa, L. H., Valladao, R., Lopes, A. R., & Oliveira, V. M. D. (2024). Genomics, proteomics, and antifungal activity of chitinase from the antarctic marine Bacterium Curtobacterium sp. CBMAI 2942.International Journal of Molecular Sciences,25(17), 9250. 10.3390/ijms25179250 10.3390/ijms25179250 PMC11395076 39273199

[CR190] Ventura-Aguilar RI, Mendoza-Acevedo S, Shirai K, Bautista-Banos S, Bosquez-Molina E, Hernández-López M (2024) Catalytic activity of chitinase encapsulated with alginate and its application in a biosensor for postharvest fungal detection. Process Biochem 146:295–303. 10.1016/j.procbio.2024.08.002

[CR191] Wang J, Chen H, Wang N, Pan X, Xia B, Xu K, Zhong B (2025a) Chitosan oligosaccharides: a natural rich, high-efficiency, and safe frontrunner for the futural fruits/vegetables preservation. Food Bioprocess Technol 18(2):1104–1124. 10.1007/s11947-024-03506-w

[CR192] Wang, J. L., Chen, Y. C., Deng, J. J., Mo, Z. Q., Zhang, M. S., Yang, Z. D., & Luo, X. C. (2023). Synergic chitin degradation by Streptomyces sp. SCUT-3 chitinases and their applications in chitinous waste recycling and pathogenic fungi biocontrol.International Journal of Biological Macromolecules,225, 987–996. 10.1016/j.ijbiomac.2022.11.161 10.1016/j.ijbiomac.2022.11.161 36403764

[CR193] Wang, J., Jiang, H., Chen, S., Li, Y., Wang, Z., Hamouda, H. I., ... & Mao, X. (2025). Mining of Novel Myrosinase with High Activity Based on Sequence and Structure Clustering for Efficient Preparation of Sulforaphane. Journal of Agricultural and Food Chemistry, 73(13), 7954–7965.10.1021/acs.jafc.5c00978 10.1021/acs.jafc.5c00978 40079554

[CR194] Wang, J., Zhu, M., Wang, P., & Chen, W. (2023). Biochemical properties of a cold-active chitinase from marine Trichoderma gamsii R1 and its application to preparation of chitin oligosaccharides. Marine Drugs,21(6), 332 .10.3390/md21060332 10.3390/md21060332 PMC10302006 37367657

[CR195] Wang, S., Fu, G., Li, J., Wei, X., Fang, H., Huang, D., & Zhang, D. (2020). High-efficiency secretion and directed evolution of chitinase bcchia1 in Bacillus subtilis for the conversion of chitinaceous wastes into chitooligosaccharides.Frontiers in Bioengineering and Biotechnology,8, 432.10.3389/fbioe.2020.00432 10.3389/fbioe.2020.00432 PMC7221128 32457893

[CR196] Wang X, Xu K, Tan Y, Yu S, Zhao X, Zhou J (2023c) Deep learning‐assisted design of novel promoters in *Escherichia coli*. Adv Genet 4(4):2300184. 10.1002/ggn2.202300184 38099247 10.1002/ggn2.202300184 PMC10716054

[CR197] Wang Y, Zhang A, Mo X, Zhou N, Yang S, Chen K, Ouyang P (2020b) The effect of ultrasonication on enzymatic hydrolysis of chitin to N-acetyl glucosamine via sequential and simultaneous strategies. Process Biochem 99:265–269. 10.1016/j.procbio.2020.09.013

[CR198] Wang ZK, Feng DT, Su C, Li H, Rao ZM, Rao YJ, Gong JS (2024a) Designing ASSMD strategy for exploring and engineering extreme thermophilic ancestral nitrilase for nitriles biocatalysis. ACS Catal 14(18):13825–13838. 10.1021/acscatal.4c03851

[CR199] Wang Z, Shen Y, Cao L, Li H, Li H, Song L, Dong C (2024b) Enhancing the catalytic activity of geranylgeranyl diphosphate synthase through ancestral sequence reconstruction and semirational design. J Agric Food Chem 72(34):19187–19196. 10.1021/acs.jafc.4c05029 39137390 10.1021/acs.jafc.4c05029

[CR200] Wang W, Liu J, Xu H, Zhang Y, Mao X, Huang WC (2024c) Characterization and comparison of carboxymethylation and TEMPO-mediated oxidation for polysaccharides modification. Int J Biol Macromol 256:128322. 10.1016/j.ijbiomac.2023.128322 38000579 10.1016/j.ijbiomac.2023.128322

[CR201] Wei H, Smith JP (2023) Modernized machine learning approach to illuminate enzyme immobilization for biocatalysis. ACS Cent Sci 9(10):1913–1926. 10.1021/acscentsci.3c00757 37901174 10.1021/acscentsci.3c00757 PMC10604017

[CR202] Wu LN, Cheng G, Jiao SM, Feng C, Li JJ, Du YG (2021) Expression, characterization, and hydrolysis product analysis of papaya chitinase in *Pichia pastoris*. Bioprocesses 19(6):8. 10.3969/j.issn.1672-3678.2021.06.007

[CR203] Wu, Q., Gong, Y., Deng, H. Y., et al. (2022). Cloning and Functional Analysis of the Promoter of Chitinase Ib ChiA from Sweetpotato (Ipomoea batatas). Molecular Plant Breeding, 20(3), 20.10.13271/j.mpb.020.000742

[CR204] Wu Q, Li HP, Liu Y, Shou C, Chen Q, Xu JH, Li CX (2024) Discovery and engineering of a bacterial (+)-Pulegone reductase for efficient (−)-menthol biosynthesis. Chemsuschem 17(23):e202400704. 10.1002/cssc.202400704 38860330 10.1002/cssc.202400704

[CR205] Xie XH, Fu X, Yan XY, Peng WF, Kang LX (2021) A broad-specificity chitinase from *Penicillium oxalicum* k10 exhibits antifungal activity and biodegradation properties of chitin. Mar Drugs 19(7):356. 10.3390/md19070356 34201595 10.3390/md19070356 PMC8307900

[CR206] Xie Y, An J, Yang G, Wu G, Zhang Y, Cui L, Feng Y (2014) Enhanced enzyme kinetic stability by increasing rigidity within the active site. J Biol Chem 289(11):7994–8006. 10.1074/jbc.M113.536045 24448805 10.1074/jbc.M113.536045 PMC3953309

[CR207] Xie WJ, Warshel A (2023) Harnessing generative AI to decode enzyme catalysis and evolution for enhanced engineering. Natl Sci Rev 10(12):nwad331. 10.1093/nsr/nwad331 38299119 10.1093/nsr/nwad331 PMC10829072

[CR208] Xu P, Ni ZF, Zong MH, Ou XY, Yang JG, Lou WY (2020) Improving the thermostability and activity of *Paenibacillus pasadenensis* chitinase through semi-rational design. Int J Biol Macromol 150:9–15. 10.1016/j.ijbiomac.2020.02.033 32035157 10.1016/j.ijbiomac.2020.02.033

[CR209] Xu, Q. Q., Zhang, Z. Y., Tao, X. T., et al. (2022). Optimization of fermentation conditions for chitinase production by endophytic Bacillus thuringiensis BT028 from Citrus aurantium. Science and Technology of Food Industry, 43(11), 8.10.13386/j.issn1002-0306.2021100183

[CR210] Xu Y, Ouyang B, Deng L, Liao M, Tang T, Lan D, Wang Y (2024) Biochemical characterization of a novel hyperthermophilic chitinase from a deep-sea Thermotogae bacterium. Process Biochem 143:60–72. 10.1016/j.procbio.2024.04.031

[CR211] Xue R, Chen Y, Rong H, Wei R, Cui Z, Zhou J, Jiang M (2021) Fusion of chitin-binding domain from *Chitinolyticbacter meiyuanensis* SYBC-H1 to the leaf-branch compost cutinase for enhanced PET hydrolysis. Front Bioeng Biotechnol 9:762854. 10.3389/fbioe.2021.762854 34976965 10.3389/fbioe.2021.762854 PMC8715031

[CR212] Yan Q, Zhang X, Chen Y, Guo B, Zhou P, Chen B, Wang JB (2022) From semirational to rational design: developing a substrate-coupled system of glucose dehydrogenase for asymmetric synthesis. ACS Catal 12(11):6746–6755. 10.1021/acscatal.2c00705

[CR213] Yang S, Fu X, Yan Q, Guo Y, Liu Z, Jiang Z (2016) Cloning, expression, purification and application of a novel chitinase from a thermophilic marine bacterium *Paenibacillus barengoltzii*. Food Chem 192:1041–1048. 10.1016/j.foodchem.2015.07.092 26304445 10.1016/j.foodchem.2015.07.092

[CR214] Yang J, Lal RG, Bowden JC, Astudillo R, Hameedi MA, Kaur S, Arnold FH (2025a) Active learning-assisted directed evolution. Nat Commun 16(1):714. 10.1038/s41467-025-55987-8 39821082 10.1038/s41467-025-55987-8 PMC11739421

[CR215] Yang, W., Wang, Y., & Wang, Y. (2025). Deep Learning-Driven Protein Structure Prediction and Design: Key Model Developments by Nobel Laureates and Multi-Domain Applications. arXiv preprint arXiv:2504.0149010.48550/arXiv.2504.01490

[CR216] Yao D, Han X, Gao H, Wang B, Fang Z, Li H, Xiao Y (2023) Enhanced extracellular production of raw starch-degrading α-amylase in *Bacillus subtilis* through expression regulatory element modification and fermentation optimization. Microb Cell Fact 22(1):118. 10.1186/s12934-023-02116-z 37381017 10.1186/s12934-023-02116-z PMC10308679

[CR217] Yin YR, Li XW, Long CH, Li L, Hang YY, Rao MD, Yang LQ (2023) Characterization of a GH10 extremely thermophilic xylanase from the metagenome of hot spring for prebiotic production. Sci Rep 13(1):16053. 10.1038/s41598-023-42920-6 37749183 10.1038/s41598-023-42920-6 PMC10520001

[CR218] Yu T, Cui H, Li JC, Luo Y, Jiang G, Zhao H (2023) Enzyme function prediction using contrastive learning. Science 379(6639):1358–1363. 10.1126/science.adf2465 36996195 10.1126/science.adf2465

[CR219] Yu P, Xu M (2012) Enhancing the enzymatic activity of the endochitinase by the directed evolution and its enzymatic property evaluation. Process Biochem 47(7):1089–1094. 10.1016/j.procbio.2012.03.015

[CR220] Yuan H, Tu R, Tong X, Lin Y, Zhang Y, Wang Q (2022) Ultrahigh-throughput screening of industrial enzyme-producing strains by droplet-based microfluidic system. J Ind Microbiol Biotechnol 49(3):kuac007. 10.1093/jimb/kuac007 35259275 10.1093/jimb/kuac007 PMC9142201

[CR221] Yuan, S. B., Chen, X. T., She, M. L., et al. (2018). Optimization of Fermentation Conditions for Chitinase Production by MEW06 Using Response Surface Methodology. Journal of Green Science and Technology, (12), 5.10.16663/j.cnki.lskj.2018.12.060

[CR222] Yuan Y, Kong D, Wu J et al (2023) Expression element optimization and molecular modification to enhance the secretory expression of chitinase from *Bacillus licheniformis* in *Bacillus subtilis*. Process Biochem 131:32–40. 10.1016/j.procbio.2023.05.029

[CR223] Yurtsever A, Wang PX, Priante F et al (2022) Probing the structural details of chitin nanocrystal-water interfaces by three-dimensional atomic force microscopy(J). Small Methods 6(9):2200320. 10.1002/smtd.202200320 10.1002/smtd.202200320 35686343

[CR224] Zakariassen H, Aam BB, Horn SJ, Vårum KM, Sørlie M, Eijsink VG (2009) Aromatic residues in the catalytic center of chitinase A from *Serratia marcescens* affect processivity, enzyme activity, and biomass converting efficiency. J Biol Chem 284(16):10610–10617. 10.1074/jbc.M900092200 19244232 10.1074/jbc.M900092200 PMC2667748

[CR225] Zhai M, Du J, Zhang J, Miao J, Luo J, Cao Y, Duan S (2020) Changes in the microstructure and enzymatic hydrolysis performance of chitin treated by steam explosion, high-pressure homogenization, and γ radiation. J Appl Polym Sci 137(48):49597. 10.1002/app.49597

[CR226] Zhang A, Wang R, Zhou X, Liu Q, Zhang X, Chen F, Chen K (2025) Genome sequence and chitinases repertoire of the *Chitinibacter* sp. strain GC72: heterologous expression and characterization of the chitinase (Chi1) for degradation of chitinous waste. Int J Biol Macromol. 10.1016/j.ijbiomac.2025.145867 40653235 10.1016/j.ijbiomac.2025.145867

[CR227] Zhang Q, Zhang X, He Y, Li Y (2023) The synergistic action of two chitinases from *Vibrio harveyi* on chitin degradation. Carbohydr Polym 307:120640. 10.1016/j.carbpol.2023.120640 36781282 10.1016/j.carbpol.2023.120640

[CR228] Zhang W, Ma J, Yan Q, Jiang Z, Yang S (2021a) Biochemical characterization of a novel acidic chitinase with antifungal activity from *Paenibacillus xylanexedens* Z2–4. Int J Biol Macromol 182:1528–1536. 10.1016/j.ijbiomac.2021.05.111 34022308 10.1016/j.ijbiomac.2021.05.111

[CR229] Zhang, X. T., Zhang, X. M., Su, Y. C., et al. (2021). Optimization of Fermentation Conditions for the Production of Chitin Deacetylase from Arthrobacter protophormiae CDA2–2–2. Science and Technology of Food Industry, 42(8), 7.10.13386/j.issn1002-0306.2020060330

[CR230] Zhang Y, Guan F, Xu G, Liu X, Zhang Y, Sun J, Tian J (2022) A novel thermophilic chitinase directly mined from the marine metagenome using the deep learning tool Preoptem. Bioresour Bioprocess 9(1):54. 10.1186/s40643-022-00543-1 38647756 10.1186/s40643-022-00543-1 PMC10991277

[CR231] Zhao Q, Fan L, Deng C, Ma C, Zhang C, Zhao L (2023a) Bioconversion of chitin into chitin oligosaccharides using a novel chitinase with high chitin-binding capacity. Int J Biol Macromol 244:125241. 10.1016/j.ijbiomac.2023.125241 37301336 10.1016/j.ijbiomac.2023.125241

[CR232] Zhao S, Liu M, Sun X, Jiang X, Li Y, Wu X, Wang L (2023b) Engineering the relatively conserved residues in active site architecture of thermophilic chitinase Ss Chi18A enhanced catalytic activity. Biomacromol 25(1):238–247. 10.1021/acs.biomac.3c00936 10.1021/acs.biomac.3c00936 38116793

[CR233] Zhao S, Li Z, Chen M, Zhu J, Jiang X, Wu X, Wang L (2025) Loop dynamics perturbation enhances the catalytic stability of GH18 family chitinases. J Agric Food Chem 73(32):20450–20461 40740023 10.1021/acs.jafc.5c04696

[CR234] Zhao S, Yan ZJ, Zhang S, Yu JH, Wu XY, Wang LS (2022) Research progress on structure, function and molecular design of bacterial chitinase. Prog Biochem Biophys 49(7):1179–1191. 10.16476/j.pibb.2021.0229

[CR235] Zhao H, Su H, Sun J, Dong H, Mao X (2024) Bioconversion of α-chitin by a lytic polysaccharide monooxygenase Os LPMO10A coupled with chitinases and the synergistic mechanism analysis. J Agric Food Chem 72(13):7256–7265. 10.1021/acs.jafc.3c08688 38438973 10.1021/acs.jafc.3c08688

[CR236] Zheng, J. M., Liang, Y. H., Zhu, F., et al. (2018). Mutagenic selection of high-yield chitinase-producing strains and optimization of fermentation conditions. Science and Technology of Food Industry, 39(9), 8.10.13386/j.issn1002-0306.2018.09.016.

[CR237] Zhou X, Chu Q, Zhang X, Chen F, Chen K, Zhang A (2025) Enhancing thermostability of a highly active chitinase Chi1 through semirational design for efficient chitin hydrolysis. J Agric Food Chem 73(36):22633–22644. 10.1021/acs.jafc.5c07458 40879565 10.1021/acs.jafc.5c07458

